# Spatiotemporal cancer control *via* alternating magnetic field -activated nanoantennae: coupling heat, electron transport, and immune reprogramming

**DOI:** 10.1080/17435889.2026.2658584

**Published:** 2026-04-16

**Authors:** Thrinayan Moorthy, Shang-Hsiu Hu

**Affiliations:** Department of Biomedical Engineering and Environmental Sciences, National Tsing Hua University, Hsinchu, Taiwan (R.O.C.)

**Keywords:** Alternating magnetic field, magnetic hyperthermia, electrical current generation, immunotherapy, solid tumor

## Abstract

Magnetic-field-responsive nanoparticles (MFR-NPs) have evolved from traditional magnetic hyperthermia agents into conductive nanomaterials that combine catalytic therapy with electrical current generation under alternating magnetic fields (AMFs). This review highlights advances in their design, showing how control over composition, size, and morphology improves heating efficiency, energy conversion, and catalytic activity. Beyond magnetic losses, AMFs can induce eddy currents and voltage gradients in conductive nanoparticles, enabling Joule heating and wireless electrochemical stimulation. These effects support controlled drug release, deeper tumor penetration, and regulation of cellular redox processes. Systems such as gold and carbon-based nanoelectrodes with redox-active biomolecules allow remote modulation of electron transport, influencing apoptosis and intracellular signaling. Magnetically triggered catalytic platforms also enhance cuproptosis and immunogenic cell death, promoting the release of DAMPs and TAAs to reshape the tumor microenvironment. Applications in glioblastoma and metastatic cancers show promise, as tailored MFR-NPs can overcome barriers like the blood–brain barrier and work synergistically with immune checkpoint therapies, offering potential for next-generation cancer immunotherapy.

## 1. Introduction

Magnetic fields have evolved over the years into a functional tool for externally stimulating magnetic-field-responsive nanoparticles (MFR-NPs) to deliver controlled and localized magnetic hyperthermia (MHT) for effective cancer therapy. Combining alternating magnetic field (AMFs) and MFR-NPs has begun a trend of transducing electromagnetic energy into thermal energy and facilitating the generation of reactive oxygen species (ROS) within solid tumors, while simultaneously sparing surrounding healthy organs and tissues. MHT mediated by iron-oxide nanoparticles (IONPs) has advanced to early clinical-stage applications, primarily in glioblastoma GBM and prostate cancer, thereby demonstrating the translational potential of MFR-NP-based regimens [1–3]. Moreover, the field has swiftly emerged and evolved beyond conventional MHT and is currently leaning toward multifunctional MFR-NPs that couple diagnostics, pharmacological delivery, and immunomodulatory effects.

At the core of this technology, involving MHT facilitated by MFR-NPs, lie the physics, mechanisms, and interactions between AMF and MFR-NPs. The generation of thermal energy by MFR-NPs depends on various factors, including magnetic anisotropy, shape and size, and nanosystem aggregation, along with Néel and Brownian relaxation, hysteresis loops, and losses, resulting in heat generation at the nanoscale [2,4]. Apart from MHT, exposure to AMF also results in mechanical vibration of nanosystems, leading to lysosomal membrane disruption and catalyzing ROS generation *via* Fenton reactions. This, in turn, activates apoptosis/ferroptosis even without the generation of a substantial amount of heat [5,6]. The sensitivity of these mechanisms can be tuned by varying material composition, surface chemistry, and crystallinity to optimize therapeutic outcomes.

Over the years, a wide variety of MFR-NPs have been designed, developed, and scrutinized. One such nanomaterial is superparamagnetic iron-oxide nanoparticles (SPIONs), which to date remain the most clinically advanced MFR-NPs. Apart from SPIONs, spinel ferrites (MnFe_2_O_4_, ZnFe_2_O_4_, and CoFe_2_O_4_) have also gained popularity and have been widely developed and scrutinized, with their magnetic anisotropy and higher specific adsorption rates (SAR) able to be altered and optimized for effective MHT under clinical conditions [7,8]. In recent times, hybrid nanoarchitectures such as Au–Fe_3_O_4_, carbon/MFR-NP composites, and metal organic framework (MOFs) have been developed with additional functionalities, including improved drug transport, photothermal effects, enhanced electrical conductivity, and catalysis [9,10]. Emerging MFR-NP platforms based on metal oxides, alloys, and core–shell structures have not only advanced in their architecture but also enabled precise and localized heating, controlled delivery of cargo, and multimodal imaging.

In cancer therapy, MFR-NPs support multiple treatment modalities to eradicate solid tumors. Conventional MHT, involving the rise of intratumoral temperatures to 42–45°C, not only triggers apoptosis but also sensitizes solid tumors to combination therapy in consolidation with radiotherapy and chemotherapy, where high temperatures achieve eradication of resistant tumors and trigger ICD [4]. MFR-NPs, when subjected to AMF, not only generate heat but also facilitate drug release from thermosensitive materials, such as polymeric and liposomal coatings and 3D hydrogel matrices, thereby enabling on-demand drug release and confining chemotherapy to the tumor microenvironment while reducing systemic toxicity [11–13]. In the recent past, magneto-catalytic nanosystems integrating iron-based cores with those of noble metals and MOFs in the exterior shell have been developed and proposed to augment ROS, radiotherapy, and chemodynamic therapy [6,9,14]. In addition to the aforementioned, MHT, in synergy with ICD, is being explored to enhance antitumor immunity and improve responses to immune checkpoint blockade. Research suggests that effective priming of dendritic cells (DCs) by MHT, mediated by antigen release, results in the effective training of helper and cytotoxic T cells for the targeting, recognition, and killing of cancer cells [15]. Moreover, in recent years, MFR-NPs have been engineered to specifically rewire the immunologic circuits of the TME to suppress the inhibitory effects of tumor-associated macrophages (TAMs) and regulatory T-cells (Tregs), thereby enabling effective infiltration of cytotoxic T cells and inhibiting cancer proliferation and progression [16].

Apart from intrinsic magnetic losses, the evolution of eddy currents has emerged as a potent engineerable concept in cancer therapy, particularly when conductive nanomaterials are exposed to AMF. Nanoarchitectures, when subjected to a magnetic field, can produce eddy currents whose amplitudes completely depend on the magnetic field strength [17]. Specifically engineered conductive MFR-NPs can harness currents to generate localized electrical/MHT perturbations. While researchers have extensively worked on modulating MFR-NPs to achieve optimal magnetic performance, a shift in research direction has been observed in recent times, with the growing importance of MFR-NP-mediated cancer immunotherapy [18,19]. For instance, conductive nanosystems, upon exposure to a magnetic field, induce eddy currents; the same occurs when coupled with mitochondrial interactions, resulting in amplification of ROS, release of damage-associated molecular patterns (DAMPs), and facilitating antigen capture to activate immunotherapy [20].

The rationale of “wireless charging” can be viewed as a link that controls intracellular bioelectronics *via* magnetic triggering. Eddy currents induced by the magnetic field can depolarize the mitochondrial membrane and further release Cytochrome-c (Cyt c), leading to apoptotic cell death [20]. The emergence of quantum signaling in biological contexts suggests that an externally applied electric field can induce electron transfer between two molecules conjugated to a conductive nanomaterial, thereby enabling quantum biological tunneling to trigger apoptosis [21]. On the whole, these significant advances position eddy-current-enabled nanoplatforms not only as heat/ROS generators but also as bioelectrical nanomodulators for desired cancer therapeutic outcomes [21].

Despite significant advancements made in the design and development of MFR-NPs for cancer therapy, various momentous challenges remain when it comes to clinical translation. Accomplishing homogeneous MHT in a clinical setup in the heterogeneous TME anchors on various factors involving biodistribution and tumor perfusion of MFR-NPs, design and development of human-sized AMF coils that are clinically safe with effective field strength and frequency [4,22]. Long-term exposure and degradation of inorganic nanoparticles, off-target heating and ROS production, and challenges associated with regulatory affairs are the current hurdles in the clinical development of MFR-NPs. The clinical results of NanoTherm® appear promising, thereby inspiring scientists and researchers to develop nanoplatforms with improved safety and reproducibility for the effective treatment of cancer [3,23].

**Figure 1.**
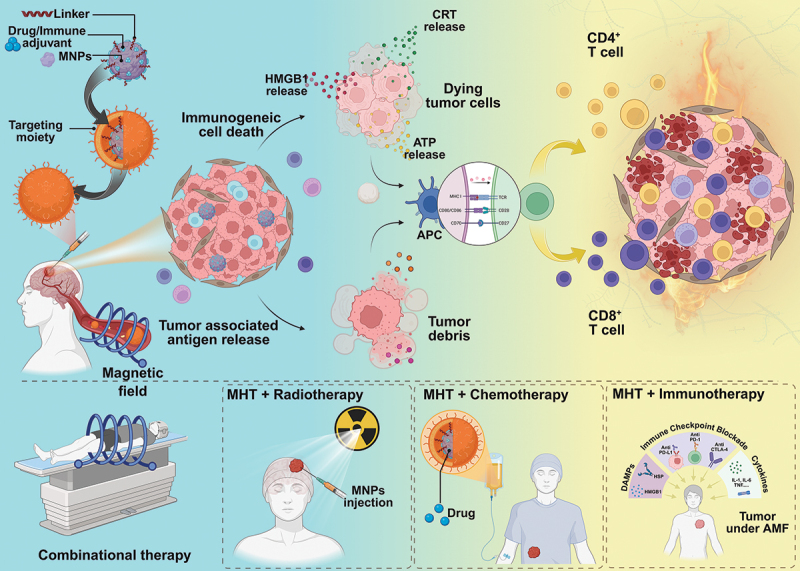
The scheme depicts the transduction of magnetic energy into therapeutic outcomes, highlighting the synergistic treatment of tumors and GBM involving thermal ablation in combination with chemotherapy, radiotherapy, and activation of the innate immune system via delivery of immune checkpoint blockade and release of tumor antigens, resulting in priming in antigen-presenting cells and T-cell infiltration. Created in BioRender. Ramesan, L. N. (2026) https://BioRender.com/8zbm3zq.

In this review, we have focused on describing a wide range of MFR-NPs landscapes for the effective therapy of cancers, along with strategies to circumvent hindrances and effectively induce ICD. Firstly, we have elucidated the physics of MFR-NPs and the underlying factors such as particle size, shape, magnetic anisotropy, and material composition, that govern hysterysis loss and ROS production—followed by the classification of AMF-responsive nanostructures involving pure metals and its oxides, ferrites, hybrid nanoplatforms, and emerging AMF-responsive nanoplatforms with emphasis on relationships between structure–property–function for cancer therapy. Based on the foundation outlined above, we elaborated and discussed the diverse therapeutic mechanisms and critical factors that enabled cancer therapy via AMF, including MHT, controlled pharmacological release, and magneto-catalytic and immunotherapeutic regimes employed. We critically reviewed and described the physiological barriers, outlined the clinical progress, and proposed future directions for precision AMF systems and multimodal nanoplatforms that combine hyperthermia with chemotherapy and radiotherapy. Apart from outcomes of combination therapy, we have primarily focused on magnetic-field-actuated nanomedicine and its design in inducing an immune response for cancer therapy *via* MHT, AMF-aided drug release, ROS generation, and sub-cellular positioning to release ICD-specific hallmarks for immunotherapy. Finally, we have elaborated on the complications and barriers associated with GBM, a highly aggressive form of cancer that requires substantial and meticulous care with respect to the design of MFR-NPs to pose cytotoxic effects on GBM tumor cells with negligible effects on surrounding healthy tissue alongside successful activation of innate immune system to reprogram GBM’s immunological niche, suppress its progression, and improve survival rates. The schematic illustration (Figure 1) depicts the functional capabilities of MFR-NPs in inducing ICD and the combination strategies employed for solid tumor and GBM therapy.

## 2. Literature search methodology

The literature cited and discussed in this review was searched and collected from reputable scientific databases, including Web of Science, Scopus, and PubMed. The articles published between January 2000 and March 2026 were primarily considered to write the review article with a strict focus on recent advancements made in AMF-responsive nanosystems over the past 5 years. While a handful of articles published in the 1990s were considered due to their relevance and mechanistic insights over magnetism. The search was made through the mentioned databases using scientifically relevant and certain keywords, phrases, and brief sentences such as “magnetic field-responsive nanoparticles,” “magnetic hyperthermia,” “alternating magnetic field,” “magnetocatalysis,” “glioblastoma,” “combination therapy,” “cancer immunotherapy,” and “cancer theranostics.” The primary aim during the search was to select research articles on the basis of novelty of material, the rationale nanomedicine design, and preference was given to the latest research and review articles that reported superior magnetic properties suitable for cancer therapy with astonishing experimental (*in vitro* and *in vivo*) outcomes with translational potential for cancer immunotherapy mediated by AMF-responsive nanomedicine.

## 3. Fundamentals of magnetically actuated nanoparticles

### 3.1. Physics of magnetic-field responsive nanoparticles

MHT is the transformation of electromagnetic energy into heat, facilitated by MFR-NPs, which occurs due to the coupling of magnetic moments (m) of atoms present within the MFR-NPs [24]. To comprehend this notion, the fundamentals of magnetic materials need to be recalled. Upon exposure to a magnetic field, the MFR-NPs’ magnetic moments align with the external field, and the degree of alignment depends on the material’s properties, the magnitude of the applied magnetic field (H), and the surrounding temperature. The magnetic moment of MFR-NPs upon exposure to ‘H’ is often measured to yield the magnetization (M) versus ‘H’ plots. These plots are called hysteresis loops, and they enable the classification of materials into ferromagnetic, paramagnetic, diamagnetic, and antiferromagnetic. The hysteresis loops can also be used to determine remnant magnetization (M_r_), coercivity (H_c_), and saturation magnetization (M_s_) [25].

The magnetic properties, such as hysteresis, of bulk magnetic matter are influenced by the structure consisting of magnetic domains, which are separated by domain walls. Nonetheless, under a particular particle size, a single magnetic domain is more stable than several domains. The composition, geometry, and domain wall energy of the material influence the specific size at which MFR-NPs form a single magnetic domain.

Apart from those mentioned above, the lattice of magnetic field-responsive materials exhibits magnetic anisotropy, rendering them highly susceptible to magnetization. The single magnetic-domain MFR-NPs have a stable antiparallel alignment with the magnetization axis and are segregated by anisotropic energy (Ea), which is directly proportional to the crystalline magnetic anisotropy constant (K) and the magnetic domain volume (V) of MFR-NPs.

(1) Ea = KV

As the size of MFR-NPs decreases, the energy required to flip the magnetic spin decreases. At high thermal energy (*k*_*B*_*T*), sufficient energy is provided to MFR-NPs for their spins to switch between energy minima arbitrarily, where *k*_*B*_ and *T* refer to the Boltzmann constant and temperature, respectively. Below a specific diameter/size, the available thermal energy is adequate to flip the direction of magnetic moments of MFR-NPs randomly, and this reversal in direction occurs continuously and spontaneously due to thermal stimulation. This effect is called superparamagnetism, in which no coercivity is observed in the M(H) plots. Under magnetic fields, the superparamagnetic MFR-NPs exhibit enormous magnetic moments with no remanent magnetic moments upon removal of the magnetic field. No hysteresis cycle could be observed in the M(H) curves of superparamagnetic MFR-NPs. The MFR-NPs exhibiting superparamagnetic effect at biological temperature (T = 37°C) are highly suitable for MHT, provided they are unreactive and inert in the absence of an external magnetic field [26].

A hysteresis is generated in both single and multi-domain magnetic materials, while the mechanism involved in heat generation is dissimilar upon exposure to AMF. As per the laws of thermodynamics and conservation of energy, the ‘H’ generates an electromagnetic field that aligns the magnetic moments of atoms, thereby generating heat in MFR-NPs while the internal energy remains constant. Representation of magnetic heating work during the magnetization cycle *via* contour integration:

(2) $$W_{\mathrm{heat}}=\oint H\cdot \mathrm{dM}$$

### 3.2. Magnetic losses, evolution of heat, and ROS

Four mechanisms work to generate thermal energy when MFR-NPs are subjected to AMF, which involve Néel–Brownian relaxation, hysteresis, eddy current, and frictional loss [26–30]. The area encircled in the hysteresis loop is directly proportional to the loss of heat upon exposure to AMF.

(3) A=∮μ0M(H)HdH

The product of hysteresis loop area (A) and frequency (f) of magnetic field oscillations gives thermal dissipation power (P), which can be denoted as *P* = A×f.

The Rosensweig model can be used to associate the properties of MFR-NPs with the area under the hysteresis loop upon exposure to an AMF [31]. The linear relationship of MFR-NPs can be denoted as M = χH. For MFR-NPs, the linear response theory (LRT) or the Néel–Brown relaxation model can be utilized upon exposure to AMF. As shown above, the product of the ‘A’ with that of ‘f’ equals ‘P,’ which is directly proportional to *H*_*max*_ and can be written as:

(4) $$P=f\times A=\mu_{0}\pi \mathrm{Hmax}2f\chi \prime \prime$$

Here, the permeability of free space has been denoted with μ_0,_ and the loss component of susceptibility (χ′′) can be further shown as:

(5) $$\chi \prime \prime =\chi_{0}[\omega \tau /(1+\omega \tau {)}^{2})]$$

The static susceptibility (χ_0_) can be further expressed as follows:

(6) $$\chi_{0}={M_{s}}^{2}V/3k_{B}T$$

The dephasing of MFR-NPs upon exposure to AMF is represented by χ′′; the magnetic relaxation time (τ) matches the period of the applied field, ωτ=2πfτ=1. The relaxation time can be represented as (1/τ)=(1/τ_N_)+1(1/τ_B_) [32], with τ_N_ and τ_B_ being the Néel and Brownian relaxation times, respectively, and are denoted as:

(7) $$\tau_{N\;=}\tau_{0}\exp\left(\mathrm{KV}/k_{B}T\right)$$

(8) $$\tau_{B}=(3V_{H}\eta )/\left(k_{B}T\right)$$

τ_0_ is the pre-exponential factor, which is a value ranging between 10^−13^ and 10^−9^ s, as it is the attempt time. The viscosity of the fluid in which MFR-NPs are dispersed is represented by η, with V_H_ being the volume. The Néel relaxation time is associated with the variation in heat, and the Brownian relaxation time is associated with the variation in rotation, where the minimal time dominates the overall relaxation duration.

It is of the utmost essentiality to describe specific loss power (SLP) for MHT. This is also referred to as the SAR and can be defined as the rate at which magnetic field-responsive materials acquire electromagnetic energy for their further conversion into heat [33]. The thermal generation posed by MFR-NPs is contingent on ‘A’ produced in the presence of AMF. This can be represented as follows:

SAR = SLP=P/ρ=Af/ρ

The Stoner–Wohlfarth (SW) model can also be employed to calculate the area enclosed by the hysteresis loop, where A = αμ_0_M_s_H_C0_. The value of α is approximately equal to ‘2,’ and H_C0_ represents the coercive field [34,35].

Apart from SAR, the intrinsic loss power (ILP) can prove to be an effective factor for the evaluation of thermal capability, which can be calculated using the following equation:

ILP = SAR/H^2^f

While MHT is mainly responsible for cancer cell death by MFR-NPs, conventional IONPs can also generate ROS and induce ferroptosis in cancer cells. This induction of ferroptosis, in combination with MHT, augments therapeutic efficiency [36]. Ferroptosis is a type of cell death involving Fe ions (Fe^2+^/Fe^3+^), which generate intracellular ROS in the presence of H_2_O_2_ *via* a catalytic process named the Fenton reaction. The reaction produces hydroxyl radicals (^•^OH), hydroxide ions (OH^−^), and superoxide radicals (O_2_^−^) by splitting H_2_O_2_, which in turn leads to lipid peroxidation and oxidative stress. This cell damage and cyclic catalytic activity cause cell death. The Fenton reaction that produces ROS is as follows:

(9) $$Fe^{2+}+\;H_{2}O_{2}\to Fe^{3+}+\;OH^{-}{+}^{∙}\mathrm{OH}$$

(10) $$Fe^{3+}+\;H_{2}O_{2}\to Fe^{2+}+H^{+}{+}^{∙}\mathrm{OOH}$$

(11) $${\;}^{∙}\mathrm{OOH}\to H^{+}{+}^{∙}{O_{2}}^{-}$$

(12) $${\;}^{∙}O_{2}+\;H_{2}O_{2}\to O_{2}+\;\mathrm{OH}\;{+}^{∙}\mathrm{OH}$$

### 3.3. Influence of magnetic anisotropy, morphology, size, and material composition

#### 3.3.1. Morphology

Nanosystems with varying morphologies have been synthesized and scrutinized for effective cancer therapy. The shape of MFR-NPs has a significant role in magnetic anisotropy for an enhanced magnetic response. The ideal approach is to alter the anisotropy of MFR-NPs to augment their response upon exposure to AMF and SAR. Magnetic anisotropy results from various factors such as shape-surface anisotropy, magnetocrystalline anisotropy (MCA), and exchange anisotropy. In MFR-NPs, the contribution of MCA is the least. Shape-surface anisotropy plays a pivotal role in soft ferrite materials [37,38]. In core-shell structures, exchange anisotropy comes into play due to the interaction between two magnetic materials with varying magnetic order [39]. Thus, tuning MFR-NPs with different shapes would result in enhanced magnetic heating efficiency for MHT in cancer therapy.

In a study, Wang et al. stated the influence of the shape and size. In their study, researchers found a commendable ILP of 221.3 nH m^2^/kg. Moreover, further modification of ferrite nanoparticles (FNP/FNPs) with organic polymeric molecules without changing the intrinsic properties would yield composites with enhanced performance for biomedical applications [40]. The MFR-NPs employed for biomedical applications usually involve nanosystems with superparamagnetic properties, with diameters less than 20 nm, resulting in reduced magnetic properties and a low magnetic heat conversion efficiency [41]. The SLP of MFR-NPs can be significantly enhanced by fabricating a core-shell structured nanosystem to adjust the K value by including hard and soft magnetic materials. The material CoFe2O4@MnFe2O4 exhibited a significantly greater power loss of 2280 W/g, which was much greater than that of CoFe_2_O_4_ (9 nm) and CoFe_2_O_4_ (15 nm) with a specific power loss of 443 W/g and 411 W/g, respectively [42]. In an intriguing research, Noh et al. and team observed that cubic Zn0.4Fe2.6O4 with a mean size of 18 nm showed greater magnetic heat conversion efficiency compared with spherical ones, attributed to its low surface spin-disorder layer ratio of 4–8% [43]. A nanoring-shaped FNPs with a vortex magnetic domain have been demonstrated to exhibit an exceptional multi-domain structure [44]. The vortex structure possesses a magnetic moment in a clockwise/anti-clockwise direction. The FNPs, when exposed to AMF, achieve a magnetic heat conversion efficiency of 1000 W/g [45]. Undoubtedly, adjusting the physical and chemical parameters of MFR-NPs would play an essential role in improving their biocompatibility and dispersability in the circulatory fluid for effective accumulation in the tumor and its therapy.

#### 3.3.2. Size

The magnetic anisotropy of MFR-NPs can also be altered by tuning the size to obtain the finest magnetic thermal conversion efficiency [46,47]. The optimal surface-to-volume ratio of MFR-NPs is the key to achieving exceptional magnetic properties [48]. The M_s_ value depends upon the size of MFR-NPs. As the size of MFR-NPs increases beyond a critical value, the M_s_ is nearly equivalent to that of bulk materials [49]. The size of MFR-NPs can be adjusted as per the required application to favor a high M_s_. The size of MFR-NPs can be varied by employing various synthesis techniques. The most commonly utilized synthesis routes are thermal decomposition, co-precipitation, hydrothermal, and polyol synthesis [50,51].

In a study assessing the effects of size, Bakoglidis et al. developed Fe_3_O_4_ nanoparticles with varying sizes and greater monodispersity [52]. The mean diameters of the spherical nanosystems were 5.1 nm, 9.8 nm, and 17.9 nm. Under the influence of a magnetic field with a strength of 20 kA/m, the spherical nanoparticle with a size of 10 nm revealed an SLP of 230 W/g. The nanoparticle-sized 13 nm exhibited a higher SLP value than 10 nm-sized nanoparticles, which can be attributed to the hysteresis loss. Thus, the SAR values of MFR-NPs are dependent on size. Lv et al. developed octahedral-shaped Fe_3_O_4_ nanoparticles with varying sizes (13, 22, 43, 98, and 260 nm) and studied the MHT effect [53]. The SAR values of nanosystems were measured at 358 kHz. The octahedral-shaped Fe_3_O_4_ nanoparticles, with sizes of 43 nm and 98 nm, exhibited SAR values of 2483 W/g and 2629 W/g, respectively. Thus, the study revealed the influence of size on thermal energy generation. Fabricating FNPs with a small size and exceptional superparamagnetic properties is a formidable task. The most commonly employed technique for fabrication is co-precipitation, due to its high efficiency and economic feasibility. In a simple yet interesting study, Dadfar et al. synthesized SPIONs *via* co-precipitation and segregated monodispersed SPIONs from polydispersed ones *via* centrifugation [54]. SPIONs with mean sizes of 7.7 ± 1.6, 10.6 ± 1.8, 13.1 ± 2.2, 15.6 ± 2.8, and 17.2 ± 2.1 nm were produced, which exhibited superparamagnetic properties with enhanced dispersion and size distribution upon comparison with the initial crude produced.

#### 3.3.3. Material composition

The material composition of MFR-NPs is of utmost importance to generate thermal energy under the influence of AMF [55]. Over the years, transition metals have gained immense popularity as they influence the M_s_ of MFR-NPs [56,57]. The most commonly used metallic ions that highly impact MHT are cobalt, zinc, manganese, and nickel. The doped transition metals highly influence the SAR value, thereby improving the overall magnetic moment [58]. Composite MFR-NPs comprising drugs, polymers, and targeting ligands can significantly affect therapeutic efficiency. In a study, Mohapatra et al. and team fabricated composite IONPs such as Fe_3_O_4_, CoFe_3_O_4_, MnFe_3_O_4_, and NiFe_3_O_4_ nanosystems *via* a technique termed thermal decomposition. The study revealed densely packed nanocrystals within IONPs, which not only caused strong exchange but also enhanced the magnetic moment. Moreover, the nanomedicine effectively killed cancer cells upon exposure to a magnetic field for 30 min [59]. In an interesting study, Sabale et al. and team fabricated MnFe_2_O_4_, ZnFe_2_O_4_, CoFe_2_O_4_, and NiFe_2_O_4_, and then calculated the SAR values of the prepared nanosystems. The research revealed SAR values of 42.26 W/g and 35.24 W/g for ZnFe2O4 and CoFe2O4, respectively, which were higher than those for NiFe_2_O_4_ and MnFe_2_O_4_ [60]. Thus, the MHT efficiency can be altered to suit the needs and biomedical applications.

While conventional Néel and Brownian relaxations facilitate nanoscale heating, engineering nanomaterials capable of generating eddy currents offers an additional benefit, as it opens a new dimension of bioelectrical activity in cancer therapy, thereby enabling a wireless modality that can circumvent the need for traditional MHT [20].

## 4. Classes of alternating magnetic field responsive nanomedicine

MFR-NPs are fabricated using metals such as Fe, Co, Ag, Ni, and Au. These metallic-nanomedicines are classified as ferrites, alloys, and metal oxides [61]. These AMF-responsive metallic nanomedicines possess superior physicochemical properties, making them highly suitable for biological applications [62]. MFR-NPs exhibit distinct features such as a high surface area to volume ratio, small size (10–200 nm), exceptional magnetic properties, and magnetic responsive imaging (MRI) responsiveness, thereby making them a suitable candidate not only for therapy but also for diagnostics [63,64].

Over the years, various MFR-NPs have been fabricated and extensively scrutinized. The MFR-NPs can be classified into pure metallic nanoparticles (Ni, Co, and Fe), metallic oxides (Fe2O3, Fe3O4, and TiO2), ferrites (MFe2O4, where M is divalent metal cations Co^2+^, Fe^2+^, Mn^2+^, Ni^2+^, Cu^2+^, and Zn^2+^), and hybrid nanosystems. The mentioned classes of MFR-NPs have their own pros and cons, and their physicochemical properties can be tuned to fit the needs of biomedical applications.

### 4.1. Pure metallic nanosystems

Magnetic field-responsive metallic nanosystems possess exceptional properties, including the presence of electrons in the outermost orbital and magnetic field responsiveness. The most widely exploited transition metals are Fe, Mn, Co, and Ni due to their superior magnetic properties [65]. Iron (Fe) based nanosystems are the most commonly used ones for biomedical applications due to their unique magnetic properties. For synthesis, Fe-based nanosystems can be readily fabricated by reducing Fe salts in desired solvents via thermal decomposition [66,67]. While the synthesis technique is practically and economically feasible, it is difficult to control during shell formation on other nanosystems. Studies have shown that meticulously designed protocols with controlled reaction factors and the use of alternate Fe precursors (such as Fe[N(SiMe_3_)_2_]_2_) produce satisfactory yields and improved size populations alongside reduced by-products [68]. Apart from Fe-based nanosystems, cobalt (Co) has also been widely studied for biomedical applications. While Fe-based nanosystems are highly biocompatible when compared with Co-based nanomedicines, results revealed significant medical benefits for Co-based nanomaterials in cancer theranostics *via* localized hyperthermia and MRI [69,70].

### 4.2. Oxidized metallic nanoplatforms and associated ferrites

Metal oxide nanomedicines stand apart from pure metallic nanoforms for biomedical applications due to their high biocompatibility, strong magnetic responsiveness, and exceptional physicochemical properties [65]. Over the years, metal oxides have gained significant attention in cancer therapy, owing to MHT, drug delivery, and surface functionalization for targeted delivery [71]. Iron (III) oxides can be classified into iron (II) oxide (wüstite), hematite, and maghemite. Iron exists in the Fe^2+^ and Fe^3+^ states, backing the construction of single-crystal phases with varied physicochemical properties. Among the various iron oxide structures, Fe_3_O_4_ exhibits semimetallic properties due to its tightly packed cubic spinel structure, making it suitable for biomedical applications [72]. Iron oxide nanosystems are easy to synthesize and yield good results under conventional synthesis techniques. Initially, orthodox fabrication protocols such as solvothermal and co-precipitation didn’t offer control over nanoparticle size and often led to aggregation, thereby hampering application efficacy. But over the years, new synthesis routes have been established to gain control over identical sizes of nanoparticles. For instance, the use of organic iron precursors yields iron oxide nanoparticles with a narrow size distribution. Moreover, tuning the concentration of surfactants and metal precursors can further aid in achieving nanoparticles with varying shapes (i.e., octahedral, spherical, and cubic) and sizes [73].

With increasing demand for biomedical applications, metal oxides were further modified to produce spinel ferrites (MFe_2_O_4_), which possess both electrical and magnetic properties [74]. This sub-division of MFR-NPs primarily comprises iron oxide doped with cationic metal ions, manganese (Mn), nickel (Ni), iron (Fe), platinum (Pt), zinc (Zn), cobalt (Co), and palladium (Pd). Doping of iron oxides with the aforementioned bivalent metallic ions significantly enhanced MHT and electrical properties, but at the same time increased cytotoxicity [71]. Thus, surface modification of ferrites with a polymer (such as polyethylene glycol (PEG)) is vital to improve biocompatibility for biomedical purposes. Various studies have synthesized surface-modified ferrites with controlled and AMF-responsive drug release, possessing low systemic toxicity [75]. Researchers have also developed bimetallic materials such as Mn-Zn and Co-Zn, and claim that increased Zn concentration decreases particle size [76]. It can be concluded that the physicochemical properties of MFR-NPs, primarily their electrical and magnetic properties, can be tuned to meet application and requirement-specific requirements by varying precursor concentration and reaction parameters.

### 4.3. Hybrid and emerging magnetic field responsive nanomedicine platforms

MFR-NPs have moved beyond pure metallic nanoparticles, metal oxides, and ferrites, with a focus on multicomponent nanostructures that not only facilitate MHT but also exert catalytic effects in ROS generation and advanced drug delivery. In recent years, magnetoplasmonic Au-Fe_3_O_4_ has gained immense popularity and has been produced in core-shell and dumbbell-shaped nanoarchitectures [77,78]. Au-Fe_3_O_4_ poses an MHT effect and acts as an MRI contrast agent due to the presence of Fe_3_O_4_, and at the same time, the presence of the Au domain also offers near-infrared (NIR) absorption and functionalization properties. As mentioned, core-shell and heterodimer structures of Fe_3_O_4_@Au and Au–Fe_3_O_4_ have shown promising synergistic therapeutic results involving MHT and photothermal therapy (PTT) [78–80]. Gold-doped/modified Fe_3_O_4_ nanostructures with unique architecture, such as star and nanopopcorn morphologies, revealed amplified photothermal conversion efficiency while preserving the magnetic property of the nanomedicine [81,82]. In a recent study, carbon-encapsulated Au/Fe_3_O_4_ nanoaggregates exhibited AMF-induced MHT and PTT, along with a π-rich surface, which, though not explored, would allow for drug loading [83].

Carbon-magnetic nanocomposites are emerging due to high colloidal stability, greater photothermal conversion efficiency, and high drug loading capacity. Carbon-encapsulated magnetic nanocomposites not only aid in achieving MHT in cancer but also act as a shield that improves stability and biocompatibility [84]. Choppadandi et al. synthesized a hybrid nanomedicine by designing amine-functionalized carbon quantum dots, which were further conjugated with MFR-NPs to obtain a nanoplatform with photodynamic capabilities while preserving MHT [85]. Reviews in recent years have highlighted carbon-based magnetic nanocomposites, including graphene, MOFs, and carbohydrate-based magnetic composites, for chemo-magnetic and magnetocatalytic therapy of cancer, which could be attributed to their high porosity, advanced surface chemistry, and photodynamic and thermal responses [86–88].

Another rapidly developing class of AMF-responsive nanosystems is MOFs. MFR-NPs are integrated with MOFs with MFR-NPs in the core and MOFs as porous shells or MOFs stand alone with decorated magnetic domains [89,90]. The porous structure of MOF facilitates loading of drugs with rich metal-ligand chemistry, which can be exploited to initiate Fenton-like reaction simultaneously with MHT, imaging for diagnostics, and tracking of accumulation of MOFs [91–93]. Magnetic field-responsive MOFs have also been shown to possess an amplified catalytic activity upon exposure to AMF, thereby enhancing chemodynamic therapy [94,95]. Apart from the mentioned, biomimetic MOFs with a platelet membrane coating on the outer surface have been studied for cancer therapy by Guo et al., which exhibit active targeting and evade immune recognition, aiding effective cancer therapy [96].

When it comes to emerging AMF-responsive nanomedicines, high-moment metallic alloys and meticulously designed core-shell structures have to be taken into consideration. Alloys such as Fe-Co exhibit a higher M_s_ than iron oxides, suggesting a higher SAR upon irradiation by AMF, leading to superior hyperthermia efficiency [34,97,98]. Strategies involving surface modification with carbon, polymers, and inert metals like Au have also been explored to embed high stability while preserving MHT [99–101]. In parallel, core-shell nanoarchitectures have also evolved, which enable photothermal ablation in combination with computed tomography (CT) and MRI for effective diagnostics [79,80,102]. On the whole, these hybrid and emerging platforms illustrate a design trend that has been moving away from conventional single-component heaters toward architected AMF nanomedicines in which magnetic loss, photonics, catalysis, and biological interfaces are co-engineered to achieve spatiotemporally programmable, multimodal cancer therapy.

The evolution of nanosystems from purely metallic to alloy to hybrid ‘nanoantennae’ clearly reflects a critical trade-off, with a shift in research directions from maximizing magnetic potential to ensuring systemic safety. Moreover, the compilation of plasmonic and catalytic interfaces enables a superior performance by allowing for multimodal and spatiotemporally programmable control over cancer therapy.

## 5. Therapeutic mechanism posed by magnetic-field responsive nanoparticles in cancer therapy

The underlying therapeutic mechanism of MFR-NPs in cancer primarily involves the transduction of AMF into thermal and catalytic cues that remodulate solid tumor biology. Magnetic materials dissipate energy via Néel and Brownian relaxation upon exposure to an AMF, thereby generating heat and magnetically induced drug release from polymers, lipids, and hydrogels, or by disturbing supramolecular gatekeepers/porous architecture [103–105]. The spatiotemporally controlled release in synergy with MHT influences cell membrane fluidity and permeability, thereby enhancing intratumoral perfusion of chemotherapeutic agents, augmenting therapeutic efficiency [106,107]. MHT facilitated by MFR-NPs can induce ICD *via* DAMPs (calreticulin (CRT), high mobility group box 1 (HMGB1), adenosine triphosphate (ATP), etc.), leading to DC activation and antigen presentation, leading to TME remodeling [106,107]. MHT also aids in relieving hypoxia, inhibiting DNA repair, and disrupting tumor vasculature, in combination with radiosensitizing agents and radiation [108–110]. Apart from MHT, the magneto-catalytic effect of MFR-NPs doped with Fe, Cu, Mn, or other metallic ions acts as nanozymes upon exposure to AMF, via a Fenton-like reaction that converts H_2_O_2_ into ROS, triggering apoptosis and redox imbalance-mediated cellular collapse [94,111,112].

**Figure 2.**
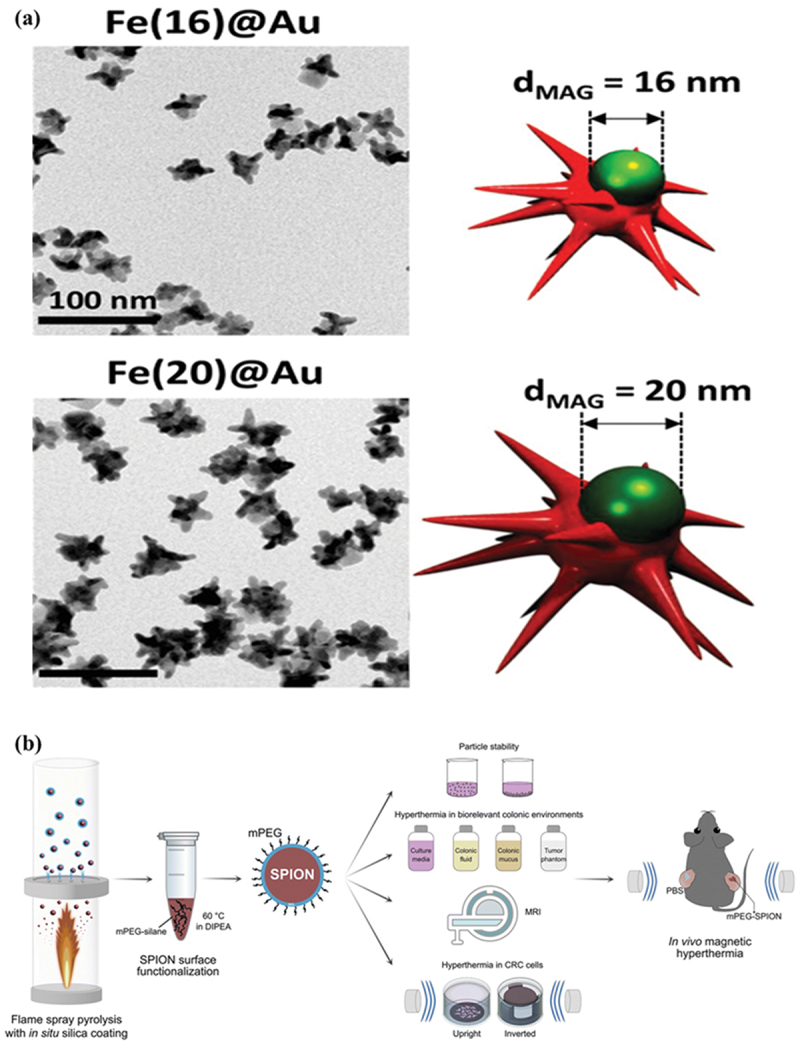
Representative nanomedicines for magnetic field-aided cancer therapy. (a) Transmission electron images and schematic illustration revealing the size of SPIONs core and spiked morphology of dual responsive (MHT and PTT) Janus gold-IONPs. Reproduced with permission from [113]. Copyright 2020, John Wiley & Sons. (b) Schematics depicting the fabrication of PEGylated SPIONs *via* spray pyrolysis of silica-modified SPIONs followed by modification with methoxy-polyethylene glycol silane for theranostics of colon cancer. Reproduced from [115], under the terms of a Creative Commons Attribution License (https://creativecommons.org/licenses/by/4.0/).

In one such study, Espinosa et al. [113] developed a Janus nanoparticle comprising a spherical iron oxide core partially covered with star-shaped nanogold, capable of excellent MHT and PTT with an optimized gold-to-iron ratio (Figure 2(a)). Apart from MHT, the particle possessed magnetic guidance capabilities, which were evaluated, and the *in vitro* results revealed significant tumor inhibition. In a fascinating study, Vergnaud et al. and team designed a bioactive nanomedicine comprising SPIONs in the core, covered by a bioglass (SiO_2_-CaO) shell for the prevention of osteosarcoma recurrence [114]. The core-shell nanosystem, γ-Fe_2_O_3_@SiO_2_–CaO, was developed *via* co-precipitation and the sol-gel technique, and bioactivity and cytotoxicity assays revealed the formation of hydroxyapatite. Zhang et al. [115] and colleagues fabricated SPIONs doped with Mn^2+^, Zn^2+^, and Gd^3+^ ions *via* spray pyrolysis, which were further surface modified with methoxy polyethylene glycol (mPEG) for improved steric stability in colonic fluids (Figure 2(b)). Apart from MHT, the nanosystem mPEG-Mn_0.6_Zn_0.4_Fe_2_O_4_ possessed diagnostic capabilities as an MRI contrast agent. The *in vivo* MHT results revealed significant damage in colorectal tumor models. On the whole, the proposed therapeutic mechanisms position MFR-NPs as multifunctional actuators that amalgamate magnetically triggered drug release, MHT, ICD, RT, and redox imbalance under a single roof for effective cancer therapy, as stated in further sections.

### 5.1. Magnetic hyperthermia aided synergistic strategies in cancer therapy

MHT has emerged as a standalone ablation strategy for sensitizing tumors, making it a potential technique for cancer therapy. By inducing heat evolution under an AMF, MHT not only facilitates drug release to enhance chemotherapy but also mediates, hinders DNA repair, and relieves hypoxia, thereby radiosensitizing tumors for effective RT [116,117]. In combination with MHT, synergistic therapeutic strategies offer an advantage in defeating cancer by overcoming dose-related cytotoxicities, effectively treating tumor heterogeneity and therapy resistance, and improving therapeutic efficacy through multimodal treatment under a single roof. The synergistic strategies employed in combination with MHT are discussed in detail in this sub-section.

#### 5.1.1. MHT in combination with chemotherapy

MHT can substantially augment chemotherapy by enhancing tumor penetration, ROS production, and increasing the cell membrane permeability of drugs released from nanocarriers, thereby facilitating drug accumulation and cytotoxicity toward the tumor while limiting systemic toxicity. MHT can sensitize tumors by disrupting protein homeostasis and metabolic pathways, pushing cancer cells toward an apoptotic threshold. Based on these principles, various AMF-responsive nanoplatforms incorporating magnetic nanomaterials with polymers, lipids, and porous inorganic drug carriers have been designed to integrate MHT with chemotherapy, and the regimes employed for cancer treatment have been summarized.

In an effort to exploit combination therapy, Fukumitsu et al. [116] engineered a synergistic strategy involving MHT and chemotherapy by incorporating various materials for drug delivery and therapy of pancreatic cancer. They effectively employed a layer-by-layer (LBL) method for the fabrication of a nanosystem, an innovative technique pitched by Decher et al. [118]. MFR-NPs were used as the core magnetic material to generate thermal energy under AMF. The MFR-NPs were further modified with a polyelectrolyte comprising gemcitabine *via* electrostatic interactions for drug delivery.

In a fascinating research leveraging subcellular organelle targeting, Shen et al. [119] reported an AMF-responsive nanosystem, abbreviated as Ir@MnFe_2_O_4_, that effectively targeted mitochondria mediated by cyclometalated iridium (III) complex. The mitochondrial glutathione (GSH) reduced Fe^3+^ to Fe^2+^, which in turn, upon reaction with H_2_O_2,_ produced ^•^OH radical due to the initiation of Fenton-like reaction redox imbalance. Upon irradiation of AMF, the Ir@MnFe_2_O_4_ nanosystem generated heat, leading to mitochondrial damage. The nanomedicine Ir@MnFe_2_O_4_ exhibited both optical and magnetic features, making it suitable for use under a photon microscope for *in vitro* imaging and responsive to MRI for *in vivo* imaging.

**Figure 3.**
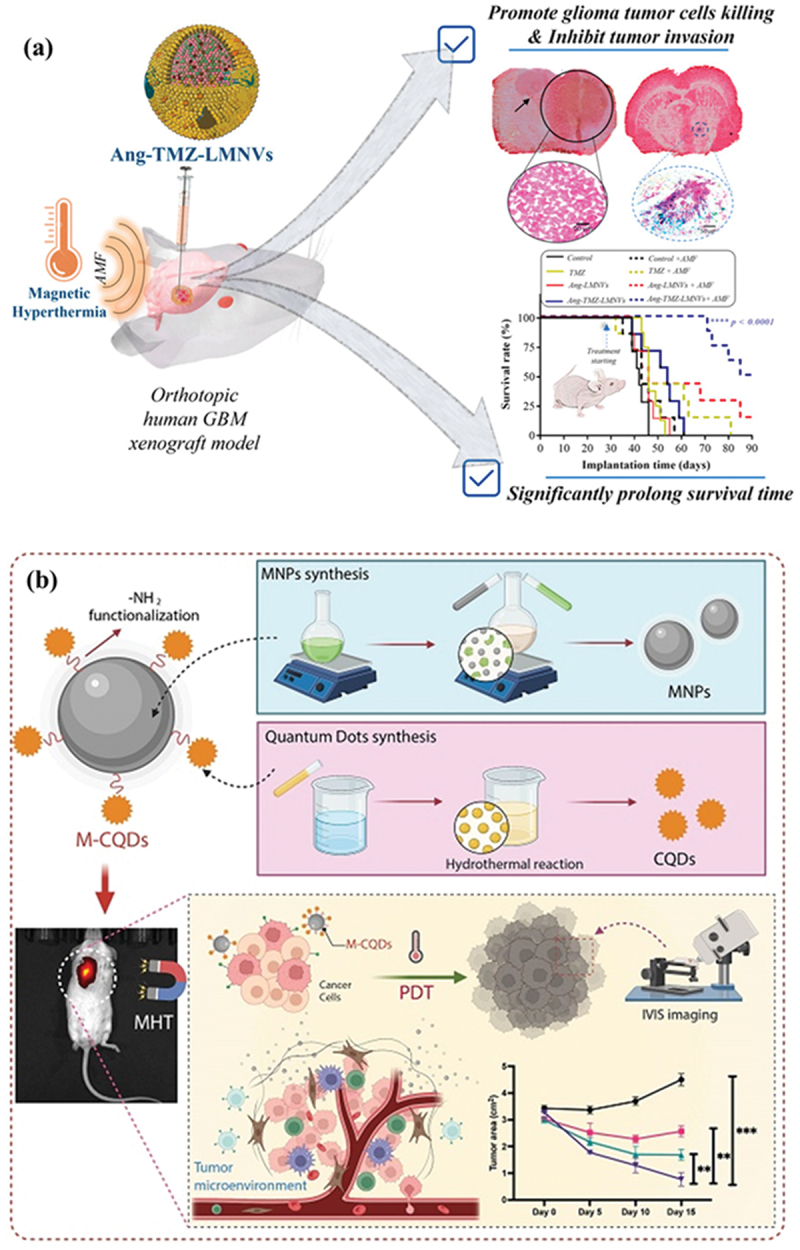
Synergistic therapy by nanomedicines, involving a combination of MHT and chemotherapy. (a) Illustration of AMF-mediated MHT in GBM by Ang-TMZ-LMNVs, associated brain tissue hematoxylin and eosin (H&E) staining revealing effective suppression of GBM, and survival outcomes in orthotopic GBM mice models Reproduced from [120], under the terms of a Creative Commons Attribution License (https://creativecommons.org/licenses/by/4.0/). (b) Schematic depiction of M-CQDs synthesis, MHT capability in synergy with PDT to kill cancer cells, and corresponding *in vivo* tumor volume curves. Reproduced from [85], under the terms of a Creative Commons Attribution License (https://creativecommons.org/licenses/by/4.0/).

Beyond cellular organelle positioning of nanosystems for effective cancer treatment, research has also focused on engineering nanomedicines with penetrative capabilities to overcome aggressive cancer forms. In one such study, Beola et al. [120] and team reported a lipid-based magnetic nanovector termed LMNVs with multifunctional properties for effective GBM therapy. The nanomedicine targeted multiple apoptotic and necrotic pathways to circumvent therapeutic resistance to enhance GBM therapy. LMNVs were reformed with angiopep-2, a GBM-targeting peptide, followed by loading with temozolomide to obtain Ang-TMZ-LMNVs. The nanoplatform effectively accumulated in GBM cells and successfully induced apoptosis mediated by MHT and chemotherapy. The therapeutic efficacy of the proposed nanosystem, Ang-TMZ-LMNVs, was evaluated in mice bearing an orthotopic GBM model created *via* intracranial injection of U-87 MG-Luc2. The multifunctional nanoplatform hindered tumor growth and extended survival when combined with MHT. Moreover, Ang-TMZ-LMNVs posed no toxicity effect on healthy tissue, as affirmed by histopathological analysis (Figure 3(a)).

Complementing penetrative strategies, Du et al. [121] designed and developed hollow Fe_3_O_4_ mesocrystals (Fe_3_O_4_ MCs) *via* a one-pot synthesis procedure involving hydrothermal reaction for the effective generation of ROS with high SAR value. The nanosystem Fe_3_O_4_ MCs exhibited a high SAR value of 722 W/g and peroxidase-like activity *via* the instantaneous generation of ^•^OH radical. The synthesis procedure employed ammonium acetate as the structural directing agent, with the reducing agent being ethylene glycol. MHT was governed by hysteresis loss with exceptional conversion efficiency when compared with Fe_3_O_4_ polycrystalline (Fe_3_O_4_ PC). This was affirmed by an increase in the area of the hysteresis loop, enhancing MHT.

The landscape of magnetic field-responsive material engineering was further extended by Choppadani et al. [85] and team, where they fabricated IONPs and modified them with amine-conjugated carbon quantum dots (QDS), abbreviated M-CQDs (Figure 3(b)). The IONPs facilitated MHT, and CQD acted as a photosensitizer to produce O_2_^−^ upon exposure to violet light. The *in vitro* anti-cancer effect of M-CQD was assessed by propidium iodide staining, accounting for 95%. The *in vivo* tumor area results further affirmed the highest anti-tumor activity with the combined therapeutic effect of MHT and PDT.

In a similar approach involving the combination of phototherapy and MHT, Ma et al. [122] reported a Fe_3_O_4_–Pd Janus nanopaltform (JNPs) capable of bimodal imaging, MRI, and photoacoustic imaging. The proposed Janus nanosystem was capable of MHT, PTT, and chemodynamic therapy. The Fe_3_O_4_–Pd JNPs exhibited a synergistic therapeutic effect facilitated by Fe_3_O_4_ (MHT) and Pd nanosheets (PTT). Both Fe_3_O_4_ nanoparticles and Pd nanosheets acted as a catalytic agent to generate ROS from intracellular H_2_O_2_ *via* the Fenton reaction. The Fe_3_O_4_–Pd JNPs exhibited an exceptional *in vivo* synergistic effect, which was assessed on orthotopic 4T1 breast cancer-bearing mice.

Substantial improvements have been made in material design for combinational therapy, which extends beyond MHT and chemotherapy to include synergistic regimens combining MHT with radiation. These regimens have proven effective in overcoming radiotherapy resistance and in triggering cancer cell death cascades in deep-seated tumors.

#### 5.1.2. MHT in combination with radiotherapy

MHT can considerably improve the outcomes of radiotherapy (RT) by promoting hypoxia relief, inducing DNA damage, and cell cycle arrest, thereby amplifying radiation-mediated cancer cell death. Beyond thermal ablation of cancer cells, the characteristics of nanomaterials, such as shape, size, and surface charge, also play a critical role in driving DNA damage. This involves direct structural and mechanical damage alongside indirect oxidative stress, resulting in single- and double-strand breaks [123]. AMF poses the potential to disrupt tumor blood vessels and to inhibit resistance against RT under hypoxic conditions without severely affecting adjacent healthy tissues. Based on these mechanisms, various nanoformulations incorporating radiosensitizing agents in combination with AMF-responsive materials have been engineered to integrate MHT with radiation therapy.

**Figure 4.**
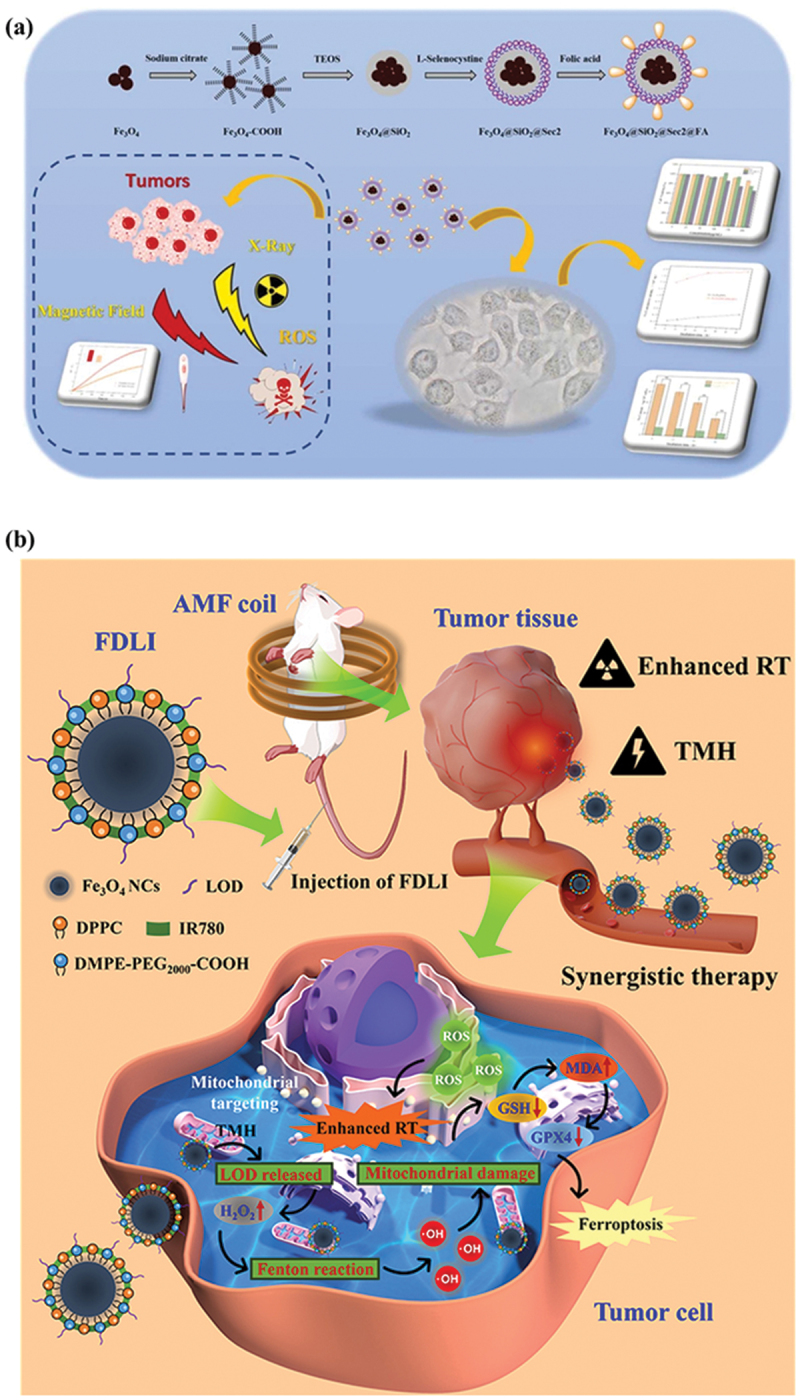
(a) Scheme represents the synthesis of Fe_3_O_4_@SiO_2_@Sec_2_@FA for targeted delivery of AMF-responsive nanoagent and radiosensitizer L-selenocystine to HeLa cells for synergistic and robust cancer therapy. Reproduced with permission from [117]. Copyright 2022, Elsevier B.V. (b) Illustration of phospholipid/PEG-coated Fe_3_O_4_ nanocomplex and its therapeutic mechanism involving a combination of targeted magnetic hyperthermia and radiotherapy, resulting in Fenton reaction, ROS elevation, mitochondrial damage, and ferroptosis. Reproduced with permission from [124]. Copyright 2026, Dove Medical Press Ltd.

In one such intriguing study exploiting MHT and RT, Lin et al. [117] proposed a multifunctional nanomedicine, abbreviated as Fe_3_O_4_@SiO_2_@Sec_2_@FA, with active tumor-targeting capabilities. The core comprises Fe_3_O_4_ nanoparticles surface-modified with a thin silica shell, followed by conjugation with L-selenocystine and folic acid (FA), which facilitates radiosensitization and targeting, respectively (Figure 4(a)). The proposed nanomedicine exhibited high M_s_, thus resulting in efficient MHT. The Fe_3_O_4_@SiO_2_@Sec_2_@FA nanosystem exhibited a clinically acceptable SAR value at a frequency of 267 kHz (magnetic field strength 18 kA/m) and 518 kHz (magnetic field strength 16 kA/m), equivalent to 57 W/g and 103.7 W/g, respectively. The FA modification resulted in ~6-fold higher uptake in HeLa cells compared with the non-targeting counterpart Fe_3_O_4_@SiO_2_, with minimal cytotoxicity. L-selenocystine present on the silica surface improved X-ray-mediated ROS generation, acting as a radiosensitizer. Combined therapeutic outcomes involving MHT and RT exhibited a cell viability of 65.9% in HeLa cells treated with Fe_3_O_4_@SiO_2_@Sec_2_@FA.

Building upon the concept of MHT-RT in targeted delivery in nanoplatforms to cancer cells, Zong et al. [124] and team further pushed the concept to a mitochondria-targeting magnetic nanoformulation Fe_3_O_4_@DPPC@DMPE-PEG_2000_-LOD@IR780 named FDLI (Figure 4(b)). A spherical Fe_3_O_4_ core was coated with phospholipid/PEG shell, followed by covalent linkage of lactate oxidase (LOD), and the NIR mitochondrial dye IR780, with the mean size of FDLI being 120 nm. Under AMF, FDLI generates hyperthermia and exhibits peroxidase-like activity. LOD transforms lactate to H_2_O_2_, triggering the Fenton reaction to produce the •OH radical by Fe ions, resulting in ROS elevation, lipid peroxidation, and activation of ferroptosis in parallel with GSH scavenging and downregulation of GPX4, thereby improving radiosensitivity. FDLI showed a high SAR value of 324 W/g with temperatures reaching 68°C. The mitochondrial targeting capability in 4T1 cells results in G2/M arrest. FDLI provides T_2_ MRI guidance, with efficient tumor accumulation and suppression as examined in 4T1-bearing mice with minimal systemic toxicity. Whereas FDL1 focuses on subcellular positioning to amplify radiosensitization, Żuk et al. [125] proposed a material-radionuclide integrated regime involving multifunctional nanoparticles with Fe_3_O_4_ as the core and ^198^Au as the shell, followed by surface modification with HS-PEG (5 kDa) and trastuzumab (Tmab) for active targeting of HER2-positive tumors. The AMF responsive magnetic Fe_3_O_4_ core aids in MHT, while the neutron-activated ^1 9 8^ Au shell provides β^−^ radionuclide therapy. The reported nanoparticles exhibited efficient heating with SAR values of 168.8 W/g (NPs-PEG) and 189.1 W/g (NPs-Tmab) and low cytotoxicity below 30 μg/mL. Tmab-conjugated NPs show specific HER2 binding in SKOV-3 cells, exhibiting enhanced cellular uptake and increased cytotoxicity at higher doses. The nanomedicine was further evaluated in 3D SKOV-3 spheroids, where radioactive nanoparticles induced significant inhibition of growth and disintegration of 3D spheroids, with amplified effects upon combination with MHT.

Completing the core-shell design for MHT-RT, Shabalkin et al. [56] and team synthesized a core – shell nanomedicine ZnFe_2_O_4_@MnFe_2_O with multifunctional properties and tunable shell (MnFe_2_O_4_) thickness capable of MRI, MHT, and RT under a single roof, aiding in theranostics. A hydrothermal reaction was employed to synthesize a multifunctional nanoplatform with varying shell thickness. Thick shelled nanoparticle (ZM3) exhibited M_s_ of 47.6 emu/g with improved dual-mode MRI contrast (r_1_ = 2.1, r_2_ = 63.4 mM^−1^ s^−1^; r_2_/r_1_ ≈30). Upon exposure to AMF, ZM3 exhibits a SAR value of 8 W/g, facilitating rapid heating up to 42°C, thereby proving to be a suitable candidate for MHT, whereas ZM0.5 exhibited negligible generation of heat. Both formulations are minimally toxic against HCT116 cells with ≈80% viability at a concentration of 160 μg/mL. As a radiosensitizer, it increased local radiation dose by 40%, thereby proving its potential as a multifunctional nanoplatform capable of MRI-guided tracking, MHT, and RT. Heo et al. [126] and team reported dextran-coated iron oxide nanoparticles (DION) that is clinically capable of MHT at 100 kHz, reaching a temperature of 43°C at a concentration of 3 mg/mL. Its therapeutic efficacy as an MHT and RT (MHRT) agent was assessed on prostate cancer cells PC3 and LNCaP, exhibiting substantial MHRT efficacy.

Overall, the aforementioned nanotherapeutic systems exert inhibitory effects on cancer cells and in animal models through synergistic effects of combination therapy. The impact of synergistic cancer therapeutic nanomedicines solely depends on their capability to sensitize resistant tumors via hypoxia relief and disruption of cancer regulatory proteins. Moreover, the thermal heterogeneity in the TME is a serious drawback that not only necessitates the development of ‘heat-free’ magneto-catalytic actuators but also immunomodulatory nanomedicines to affirm durable clinical outcomes. The TME is complex, with various inhibitory cells (TAMs and Tregs) that secrete a cocktail of cytokines to counteract the therapeutic effects of MHT, chemotherapy, and radiotherapy, and that are constantly evolving. Thus, the immunosuppressive environment not only reduces therapeutic efficacy but also continuously exerts chemokine forces, resulting in mutation and heterogeneity. To overcome these barriers and complications, researchers have been developing immunotherapeutic MFR-NPs that not only stress cancer cells *via* MHT and chemodynamic activity but also trigger immune cascades to effectively activate the innate immune system.

## 6. Rationally designed next-generation AMF-actuated nanoarchitectures for cancer therapy: immuno-oncologic relevance

The recent advancements in AMF-responsive nanosystems have gone beyond MHT to develop nanomedicines capable of activating immunological cues, confining hyperthermia, and releasing cargo on demand while accelerating redox imbalance to remodel the tumor-immune interface. While MHT by itself exerts cytotoxic effects, it also modulates and promotes ICD, thereby supporting DC priming and stimulating antitumor immunity [127,128]. The “next-generation” design advancements primarily focus on: (i) biofunctionalized hybrid nanoarchitectures that enable preferential accumulation and retention in the tumor alongside active engagement with immune cells; (ii) AMF-triggered cargo release involving magnetothermal pharmacokinetics, and (iii) Magnetocatalytic effect to enhance ROS that complement MHT for effective cancer cell death [129–132]. Finally, cancer cell organelle-damaging nanosystems, notably nanoagents that depolarize mitochondria, have paved the way for nanomedicine-aided immunotherapy in oncology [133,134]. The section ahead presents ICD-inducing next-generation MFR-NPs and the design toolkit that has been explored and exploited for immuno-oncologic intent.

### 6.1. Biofunctionalized hybrid MFR nanomedicines

Biofunctionalization is a technique that has been utilized to decorate magnetic-field responsive cores with targeting ligands (e.g., peptides/antibodies) and cell membranes as stealth coatings to improve blood circulation, achieve effective tumor homing and internalization, thereby fulfilling the key requisites for efficient anti-tumor effect, MHT, and engagement of immune cells under AMF. Such biohybrid nanoconstructs enable effective interaction with the biological interface to maximize MHT’s ability to activate the innate immune system [135–138].

Thus, in order to meet these requisites, Wang et al. [139] engineered a nanosystem capable of targeting cancer cells and remodeling the TME by converting TAMs into an M1 phenotype. They synthesized PB, which was calcined in air to yield porous, cubic magnetic iron oxide nanoparticles (IONPs). The IONPs were coated with the cell membrane of M1 macrophages derived from lipopolysaccharide-activated RAW264.7 cells and named the lung metastatic colorectal cancer intervention IO@MM. The macrophage membrane-coated IO@MM comprises proteins that enable targeting capabilities. The nanocarrier IO@MM was designed to deliver toll-like receptor 7 and 8 (TLR7/8) agonist resiquimod. The therapeutic regimen involved exposure to a magnetic field, leading to the MHT effect posed by IO@MM on CRC-bearing mice, which transformed cold tumors into hot tumors. The flow cytometry results revealed enhanced *in vitro* cellular uptake of IO@MM by CT26 cells when compared to IONPs. The biodistribution assay showed notable accumulation of IO@MM in the metastatic lungs model by circumventing immune clearance. The *in vivo* immunohistochemical quantification results showed a significant increase in the numbers of CD4^+^ and CD8^+^ cells, to 55.19 and 34.18, respectively, in the IO@MM+resiquimod-treated group.

**Figure 5.**
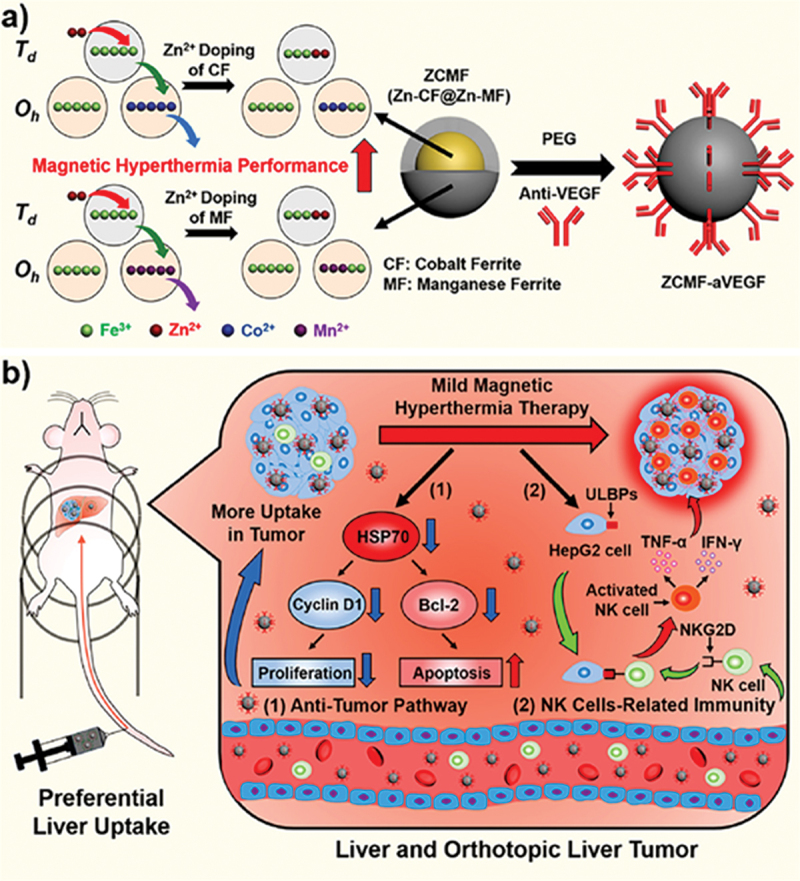
Schematic illustration of core-shell nanosystem ZCMF-aVEGF synthesis and its active-targeted delivery in liver tumors for proliferation inhibition *via* mild-MHT and activation of NK cells in orthotopic liver tumor-bearing mice. Reproduced with permission from [140]. Copyright 2021, American Chemical Society.

In addition to macrophage membrane-enabled targeted delivery of MFR-NPs, researchers have employed receptor-mediated targeting *via* antibody conjugation to the nanosystem surface. In one such fascinating report, Pan et al. [140] and team developed an intravenously injectable nanotherapeutic system for the treatment of orthotopic liver tumors *via* mild-MHT and ICD (Figure 5). The mild hyperthermia nanoparticles ZnCoFe_2_O_4_@Zn-MnFe_2_O_4_ nanoparticles generated thermal energy via exchange-coupled magnetism existing amongst the solid CoFe_2_O_4_ core and the soft MnFe_2_O_4_ shell. The MFR-NPs were further conjugated with anti-vascular endothelial growth factor (aVEGF) for effective targeting of VEGF overexpressed on HepG2 cells (hepatoma cells), and this was termed ZCMF-aVEGF. The results revealed a drastic reduction in proliferation and triggering of apoptosis in HepG2 cells under mild MHT. Moreover, mild MHT led to aberrant elevation in the expression of UL16-binding proteins and ligands of NK group 2 member D, in HepG2 cells, signifying stimulation of NK cells. In orthotopic liver tumor-bearing mouse models, a notable inhibition of tumor growth was observed, with an extended survival of more than 80 days.

In an interesting study involving the incorporation of membrane-bound particles released from cells, Shen et al. [141] and team developed an exosome-cloaked iron-oxide nanocore with high doxorubicin (DOX) payloads and coined it nanoraspberry (RB-Dox@Exo) for targeted therapy of a metastatic lung cancer model. The nanosystem RB-Dox@Exo exploits the less-explored phenomenon of nanoparticle-triggered extracellular leakiness (nanoEL), which disrupts cell–cell junctions, thereby improving the nanomedicine’s penetration and immune access. The study carried out *in vitro* assays on B16F10 melanoma cell lines, with a notable penetration in the spheroid structure upon treatment with RB@Exo. The magneto-responsiveness of RB-Dox@Exo opens up a combinational therapeutic landscape involving thermal chemotherapy that significantly elevated T-cell infiltration (CD8^+^) in the tumor, alongside higher fractions of CD4/CD8 as assessed by flow cytometry. Most importantly, mice treated with RB-Dox@Exo+AMF showed a remarkable survival rate of ∼80%. The therapeutic efficiency was mainly concluded to be from both the margination effect leading to effective accumulation of the therapeutic nanoagent, and AMF-aided DOX release at the lung metastasis.

In addition to antibodies for receptor-mediated targeted delivery, naturally occurring polysaccharides, such as hyaluronic acid (HA), have been extensively exploited for active targeting. In one such study, Qi et al. [142] and team designed a novel nanoformulation for mild-MHT in bladder cancer, with results revealing effective activation of the innate immune system. The researchers synthesized Zn–CoFe_2_O_4_@Zn–MnFe_2_O_4_ nanomedicine (MNP) with a mean size of 17 nm, followed by coating with HA to embed targeting capabilities by adhering to the receptor for hyaluronan-mediated motility. The MHT temperatures were in mild ranges between 43 and 44 ^◦^C upon exposure to AMF, resulting in polarization of TAMs into M1 phenotype *via* the TLR4/MyD88/NF-κB p65 pathway in parallel with adaptive immune response involving CD-8^+^ T-cell activation *via* DC maturation.

In a study that stands apart from usage of cellular components cloaked nanosystems for camouflaging and targated delivery, Huynh et al. [143] and team reported a multi-grained iron oxide nanoparticles (MIO/MIOs) of nearly 150 nm diameter adsorbed onto red blood cells (RBC/RBCs) for effective hitchhiking of MIOs to lung metastasis. The therapeutic system was named MIO@RBC, which, upon exposure to AMF, led to cancer cell lysis to promote the release of neoantigens and DAMPs, which were in turn captured by MIO and delivered to the lymph node, resulting in the maturation of DCs and activation of cytotoxic T-cells. Superconducting quantum interference device employed to determine magnetic flux, the results revealed a smooth curve and the MIO (0.8 mg/mL) effectively reached a temperature of ~50°C within the first two minutes of AMF (4 kA/m, 50 kHz) exposure. The cytotoxicity assay carried out on B16F10 melanoma cells revealed cell viability of nearly 80% across MIO concentrations, but it dropped to ~60% for MIO@RBC at 20 µg/mL, which can be attributed to higher cellular uptake, as confirmed by confocal images. The live/dead assay carried out on 3D tumor spheroid revealed effective cell death. The mice bearing lung metastasis treated with MIO@RBC+AMF showed a significantly reduced number of tumor foci (~50) with elevated levels of inflammatory cytokines (IL-2 and IL-12) and effective infiltration of T-cells (CD4^+^ and CD8^+^).

### 6.2. AMF-Governed on-demand pharmaceutical agent release

The idea behind the design of AMF-triggered drug release is to decouple drugs from magnetic cores upon irradiation of the magnetic field at the tumor site, thereby limiting systemic exposure while the nanoarchitecture is in the circulatory system [144]. Also, the central logic is to address the limitations of nanomedicine-aided cancer therapy, where even with effective tumor accumulation, off-target drug leakage and asynchronous dosing can hinder therapeutic efficiency and further complicate combination therapeutic regimens. The rationale behind AMF-triggered cargo release naturally aligns with cancer immunotherapy, as it can be programmed to overlap with the time window of antigen availability/release, DC maturation, and T-cell activity [129].

One such study reported by Hsu et al. [145] involves a DNA-architected nanoraspberry depot (DNR) capable of releasing Dox under a magnetic field and capturing intravenously injected Dox from the circulatory system to refill the nanoraspberry depot *via* Dox-DNA affinity. The DNR showed rapid heating with a temperature of 60°C attained within 1 min of exposure to the magnetic field. On-demand drug release was assessed by 2-min irradiation of magnetic-field, which resulted in the burst release of more than 50% Dox from DNR. The subcutaneously injected GBM mouse models showed a significant reduction in tumor volume in the DNR-treated groups combined with a magnetic field upon Dox refilling. Eminent levels of CD4^+^ (7%) and CD8^+^ (29.6%) cells were observed in DNR-treated groups in combination with a magnetic field.

Building on the concept of refillable AMF-actuated depot, the nanosystem design has shifted to multi-mechanistic models involving on-demand drug release to deliver chemotherapeutic drugs and inhibitors for immunomodulatory effects. In research guided by this rationale, Chen et al. [146] and colleagues proposed a nanoformulation, Nano@AGBH, a magnetic field-responsive nanodrug that combines AMF with starvation, chemotherapy, and an ectonucleotidase inhibitor to augment the antitumor effect. The core comprises iron oxide nanoparticles with a M_s_ of 67.5 emu/g, which were sequentially loaded with glucose oxidase and L-buthionine sulfoximine to deplete glucose and GSH, respectively, and coated with hyaluronic acid for effective tumor accumulation. Upon exposure to AMF, the nanoarchitecture reached temperatures of nearly 50°C to attenuate immunogenic cell death and ATP release. The ectonucleotidase blocker (ARL67156) not only hindered ATP to adenosine conversion, but also aided in sustained immune response in breast cancer-bearing mice models. The formulated nanomedicine (Nano@AGBH) in combination with AMF and anti-PD-L1 elevated mature DCs upto 64.9%, with CD4^+^ and CD8^+^ cells accounting to 54.4% and 20%, respectively. Moreover, a 60% survival rate was observed in tumor-bearing mice over 105 days.

Complementing the delivery of immunomodulators governed by magnetic fields, nanoarchitectures have been specifically designed to exploit vascular margination and enrichment at metastasis, where Chiang et al. [147] and team fabricated a mesoporous donut-shaped magnetic nanoparticle (pDox@donut) loaded with poly(L-glutamic acid)–doxorubicin (pDox) for targeted therapy of lung metastasis *via* the margination effect, which preferentially accumulates in the tumor. Under the influence of the magnetic field, the pDox@donut burst released pDox, and at acidic pH, the hydrazone linkages on the pDox particles were cleaved to release Dox. Under a magnetic field of 50 kHz frequency, the nanoparticles generated a temperature of nearly 60°C within 3 min. About 25% of Dox was released at a pH of 5.9 upon 2 min exposure to a magnetic field. Mice bearing a metastatic lung cancer model induced by injecting melanoma cell line (B16F10 cells), treated with pDox@donut in combination with a magnetic field, showed a remarkable reduction in tumor foci and infiltration of CD4^+^ and CD8^+^ T-cells in the tumor, as affirmed by immunohistochemical staining of lung tissue.

**Figure 6.**
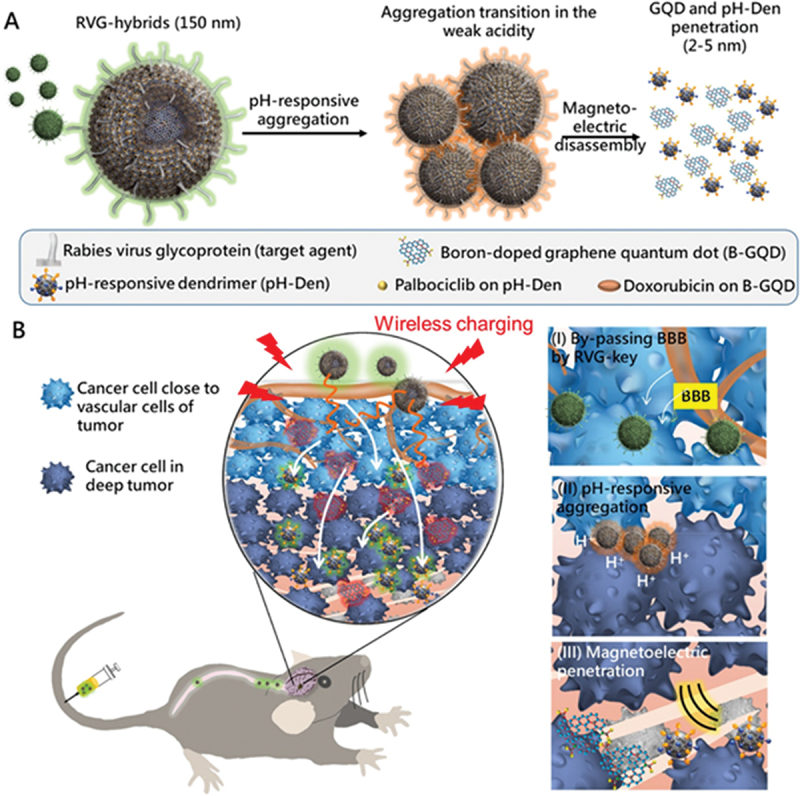
Illustration representing magnetoelectric disintegration of RVG-hybrids upon aggregation in weakly acidic conditions, their intravenous delivery *via* neural pathway, targeting of GBM, and *in vivo* magnetoelectric distortion of RVG-hybrids for deeper penetration of the drug in the tumor. Reproduced with permission from [148]. Copyright 2020, Elsevier B.V.

Apart from shape-mediated enrichment of nanomedicines in cancer tissue, MFR-NPs have been engineered with ligands for deep tumor penetration in anatomically restrictive sites for which Su et al. [148] developed a palbociclib and DOX-loaded boron-doped graphene quantum dots covered by G2 polyamidoamine dendrimer and decorated with rabies-virus-glycoprotein (RVG) for amplified treatment and accumulation in orthotopic GBM-bearing mice (Figure 6). The proposed nanosystem, named RVG-hybrids, disintegrated under a magnetic field due to magnetoelectric currents, thereby inducing weak hyperthermia and enhancing penetration and drug release. An effective release of DOX and palbociclib was observed at a pH of 6.8 upon exposure to a magnetic field for 15 min. The results of 3D spheroid cellular assays carried out on ALTS1C1 cells showed that cells treated with RVG-hybrids in combination with a magnetic field were effectively killed, resulting in 65% cell death. The signals of RVG-hybrids in the brain tumor were reduced 14 days post injection, which was attributed to the result of the immune response in the brain, as affirmed by IBA1 staining for microglia/macrophage cells.

### 6.3. Magnetocatalytic ROS augmenting nanomedicine coupled with MHT

Magnetocatalytic nanomedicines exploit the biologically available H_2_O_2_ at TME to generate cytotoxic ROS *via* a Fenton-like reaction, but the major constraints in cancer are the antioxidant effect (by GSH), the expulsion of nanomedicine, and limited ROS-producing substrates, which hinder therapeutic efficacy. Thus, coupling AMF-driven MHT provides a dual-mode synergistic effect through localized heating, accelerating redox kinetics to induce proteotoxic stress, thereby limiting the need for a heavy thermal dose for tumor control and preserving the integrity of surrounding healthy tissue. The convergence of catalytic effect and MHT, posed by a single nanoagent, has increasingly been positioned as a programmable field of nanomedicine to augment oxidative stress in cancer cells, subsequently downstream immunogenic signaling [2,93,112].

In an effort to systematically trigger the generation of ROS, Yuan et al. [149] meticulously designed an orally administrable CuFe_2_O_4_/SrTiO_3_ (CFO/STO) thermoelectric heterojunction nanosystem that was camouflaged with a fused membrane of S. aureus and macrophage for effective targeting and accumulation in orthotopic colon cancer. Under the influence of AMF, the CFO drives the magnetothermal effect, while the STO transduces the mild temperature change into charge separation to facilitate the magnetocatalytic effect, generating anionic superoxide (•O_2_^−^) and hydroxyl (•OH) radical. The oral dosage employed was 5 mg/kg, followed by AMF (3 kW, 480 kHz) after 12 hours. The fusion membrane-cloaked nanosystem (CFO/STO@M) in combination with AMF elicited an immunological response, with TAMs (24.7→7.91%) being repolarized into M1 macrophages (14.7→37.8%). The therapeutic regimen increased *in vitro* DC maturation to 48.4% and elevated intratumoral CD4^+^/CD8^+^ levels compared with controls. In a recent study to amplify redox activity, Chiang et al. [150] reported a dual-catalytic oxide nanosponge, termed DON, engineered from calcined Purssian-blue (PB) as the base substrate, followed by coating with CeO_2_ to yield a cubic nanostructure. The nanotherapeutic agent combined magnetic-field-aided chemodynamic therapy (CDT) with autologous antigen capture for immunotherapy of lung metastasis. The nanomedicine DON was characterized, and magnetic hysteresis confirmed its superparamagnetic nature, with an Ms of 16.7 emu/g. Under the influence of a magnetic field, DON accelerated Fe^3+^ /Fe^2+^ and Ce^3+^ /Ce^4+^ redox reactions to produce hydroxyl radicals (•OH) and simultaneously depleted GSH. The mice bearing lung metastasis showed a reduction in nodules from 800 to <160, with an increase in mature DC (34.3%) compared to the control (10.2%) and enhanced T-cell infiltration in the lung metastasis.

**Figure 7.**
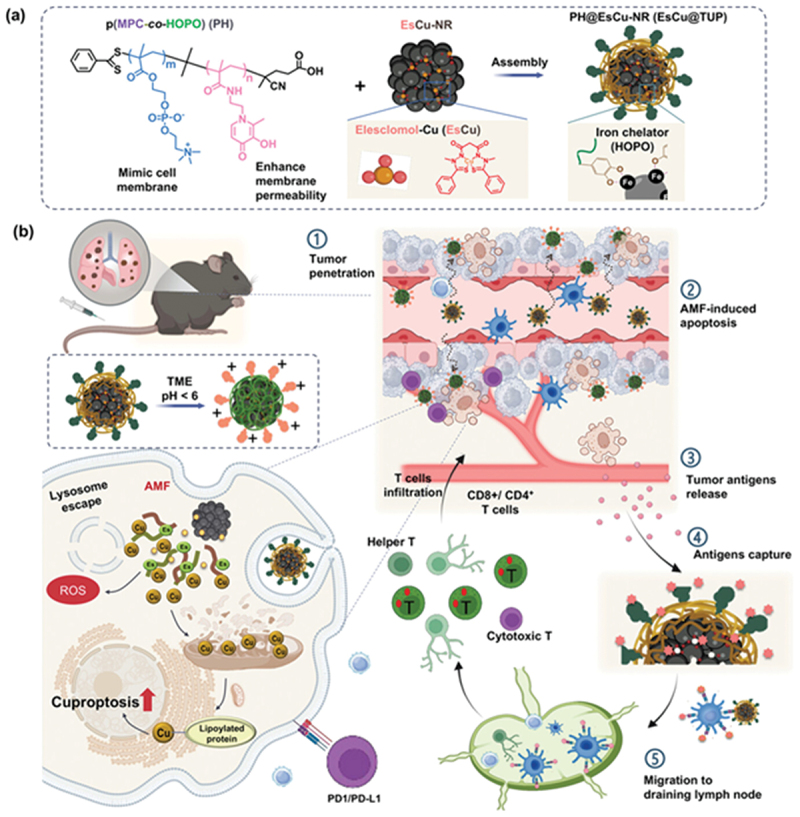
Scheme depicting the fabrication of tumor-penetrating EsCu@TUP and AMF-mediated MHT and EsCu release to actively induce cuproptosis and apoptosis, resulting in antigen presentation and capture followed by activation and infiltration of T-cells in the lung metastasis, leading to ICD. Reproduced with permission from [151]. Copyright 2024, John Wiley & Sons.

To further expand research and therapeutic outcomes of magnetocatalytic effect, Huynh et al. [151] and team synthesized a magnetic nanoraspberry (Fe_3_O_4_, NR) coated with a cell-membrane mimicking copolymer (MPC-co-HOPO, PH) encapsulating elesclomol-Cu (EsCu) to obtain EsCu@TUP for effective immunological programming of lung metastasis (Figure 7). The PH coating enables antifouling circulation, pH-mediated charge conversion, and enhances cell uptake and penetration in the 3D spheroid model. The HOPO segment, consisting of hydroxypyridin groups, facilitated antigen capture, thereby serving as an antigen reservoir to prolong immune system activation. The characterization tests revealed significant heating upon AMF and an Ms of 81 emu/g. The Cu chelated by elesclomol drives cuproptosis and mitochondrial ROS. The mice bearing metastatic lung cancer treated with EsCu@TUP+AMF showed a 150% increase in CD8+ T cells compared to the NR-treated group, along with significant DC maturation, and an extended survival was observed in synergy with αPD-1.

MFR-NPs not only pose therapeutic benefits *via* MHT but can also be engineered to aid tumor-specific accumulation through magnetic targeting. In one such study, Zhao et al. [152] and colleagues synthesized a yolk–shell nano hybrid architecture (FCMP) consisting of Fe_3_O_4_ spherical interiors covered by C/MnO_2_ shell and p53 plasmid, abbreviated as FCMP/p53, capable of reprogramming TME and activation of innate immunity. FCMP exhibited remarkable catalytic activity to deplete GSH and generate •OH radicals, as affirmed by the degradation of methylene blue. The nanohybrid therapeutic modality polarized TAMs into M1 macrophages alongside DC maturation to trigger ICD *via* CDT and PTT. The FCMP/p53 acted as an MRI contrast agent, facilitating image-guided therapy. The chemodynamic therapy in combination with PTT facilitated the release of TAAs to activate the immune response.

The TAAs are released in a burst from cancer cells during MHT and are subjected to dilution, proteolysis, and rapid clearance *via* interstitial/lymphatic drainage and phagocytic uptake. This was countered in research by Yalamandala et al. [153], where researchers developed a catalytic nanogel termed nano-reservoir (CN) capable of MHT and inducing ICD *via* antigen capture. The AMF-responsive nanotherapeutic system was fabricated from calcinated Prussian blue (PB) nanoparticles which was coated with poly (N-isopropylacrlamide)/polydopamine (PNIPAAM/PDA) comprising manganese oxide throughout its lattice. The proposed therapeutic modality, CN, exhibited MHT and chemodynamic effects, which led to the induction of apoptosis, resulting in the discharge of TAAs. The Mn ions in the nanostructure augmented catalysis. CN efficiently targeted the lung *via* marginated targeting and phagocytic cell uptake. The CN consisted of catechol groups arising from PDA, which facilitated TAAs arrest allowing for sustained release of antigens for prolonged stimulation of the immune system. CN treatedposed a robust anti-metastatic activity in BALB/c mice which was affirmed by a decrease in the number of tumor foci (<10) when compared with control (~440 tumor foci) group. Immunohistochemical staining of tumor sections revealed augmented infiltration of CD^8+^ T cells. Moreover, the flow cytometry analysis affirmed a robust immune response in metastatic lung cancer tissue accounting to 58% and 38.3% of CD^4+^ and CD^8+^ T cells, respectively. The survival curves revealed a 50% survival rate over a period of ~60 days for mice treated with CN + AMF + αPD-1 thereby proving to be an effective anti-metastatic agent.

With significant advancements made in the design of MFR-NPs to exploit MHT mediated ROS generation and ICD the concept was further advanced by combining phototherapy with for which Yilmazer et al. [154] synthesized multimodal bismuthene nanosheets for lung cancer immunotherapy mediated by PTT, MHT, and ferroptosis. The bismuthene nanosheets were fabricated by exfoliation of multi-layered bismuth (Bi) synthesized *via* surfactant-assisted chemical reduction for enhanced magnetic properties. The bismuthene nanosheet exhibiting PTT and MHT, which synergistically enhanced intracellular ROS (•OH radicals), cancer cell death as experimented *in vitro* and *in vivo*. The nanosheet-treated groups translocated calreticulin, resulting in the activation of DCs and cytotoxic T-cells.

The rationale for oxidative stress-mediated cancer cell death is not limited to ROS production; in recent years, researchers have extensively studied the production of reactive nitrogen species (RNS) for the effective killing of cancer cells. In one such study, Hu et al. [155] and team reported a Hierarchically decorated MFR-NPs (MNPs-SS-R-GOx) comprising an inner disulfide layer that mediates GSH depletion, followed by a layer of L-arginine, and an outer layer of glucose oxidase (GOx). The nanosystem is named MNPs-SS-R-GOx. aided in amplified oxidative stress due to enhanced intratumoral penetration capabilities. The GOx embedded in the nanosystem further elevated H2O2 levels to acidify and enable Fe-mediated Fenton chemistry to produce ROS (·OH radical) and simultaneously produce NO from arginine, which yielded ONOO^−^. The designed nanomedicine exhibited elevated levels of ROS/RNS signals, as affirmed by TMB (3,3′,5,5′-tetramethylbenzidine) chromogenic assay and DCFH-DA and DAF-FMDA probe in 4T1 cells. Moreover, MNPs-SS-R-GOx-treated 4T1 cells showed a ~ 30% decrease in cell viability at a minute concentration of 5 µg/mL. Under the influence of AMF (15 kA/m, 300 kHz), the intratumoral temperatures of mice treated with MNPs-SS-R-GOx reached ~47°C, significantly improving 50-day survival to 80%. The immune system remodeling was affirmed by repolarization of TAMs M2→M1 *via* markers of iNOS-positive cells (86%) and CD206-positive cells (9%), along with high T-cell infiltration in the tumor tissue (CD4 + 14.39%, CD8 + 26.93%).

### 6.4. Organelle targeting AMF-Responsive nanosystem in immunogenic signalling

The cellular organelle targeting strategy has been used to stress-specific tumor cell components to induce ICD. This involves targeting and stressing organelles such as mitochondria, lysosomes, and the endoplasmic reticulum (ER), thereby triggering the release of damage-associated signals, including Cyt c, ATP, and HMBG1, and the translocation of CRT, resulting in priming of DCs and downstream T-cell immunity [156,157]. Compared to just bulk heating of the tumor, MHT can trigger a robust immune response and ICD *via* the release of aforementioned immunogeneic cues. Recently, organelle-centric studies of tumors have revealed that positioning MHT-inducing nanoagents at the subcellular level amplifies *in vivo* apoptotic and pyroptotic signals, thereby mediating the activation and infiltration of DCs and T cells, respectively [158]. Additionally, lysosomal-targeting systems have been shown to induce lysosomal membrane permeabilization (LMP) driven ICD, convert cold tumors into hot ones, and produce effective therapeutic effects in synergy with anti-PD-L1 [159].

In a fascinating research focusing on the subcellular positioning of nanosystems, Zhang et al. [158] and team designed ferrimagnetic vortex-domain iron oxide (FVIO) nanorings and tuned surface chemistry with 3,4-dihydroxyhydrocinnamic acid (DHCA) to obtain FVIO-DHCA. The colocalization capability was assessed to reveal effective lysosomal localization with a Pearson’s coefficient value of 0.77 for FIO-DHCA, whereas FVIO surface modified with a cell penetrating peptide (CPP) largely accumulated into the cytoplasm with a Pearson’s coefficient of 0.06. The IC_50_ of FIVO-DHCA in combination with AMF accounted for 46.28 µg/mL, which was much lower than the IC_50_ of FIVO-CPP+AMF (76.95 µg/mL), thus proving lysosomal targeting to be effective. Hepa1-6 cells treated with FVIO-DHCA+AMF exhibited greater ICD hallmarks, CRT and HMGB1, and the *in vivo* results correlated with the same. The proposed tumor vaccine, capable of lysosomal-targeted MHT, markedly increased mature lymph node DCs, resulting in T-cell infiltration (CD4+ and CD8+) that was 1.8 and 1.5 times greater than that observed with the cytoplasmic-localized MHT therapeutic regimen.

In a notable study with a similar rationale, Wei et al. [160] reported a selective-targeting strategy that induces mechanical stress on cancer-cell lysosomes, thereby releasing Fe^2+^ ions to mediate a Fenton-like reaction aided catalytic cancer-cell death. The engineered nanosystem was designed with cubic magnetic nanotorquers as the core, followed by SiO_2_ coating, and surface modified with the targeted agent T7 peptide for effective delivery to breast cancer cells. The resulting nanomedicine was termed as T7-MNTs, with well-defined morphology and M_s_ of 84.97emu/g alongside pH-dependent iron release. The nanosystem T7-MNTs preferentially accumulated into lysosomes as affirmed by bio-TEM, confocal imaging, and Manders coefficient in MCF-7 and MDA-MB-231 cells. Upon exposure to rotating magnetic fields, the torque produced by T7-MNTs disrupted the lysosomal membrane, releasing endogenous Fe^2+^, leading to ferroptotic damage. Importantly, immune-cell phenotyping, such as DC maturation, T cell infiltration, and repolarization of TAM, though not assessed, the immunohistochemical staining revealed downregulation of GPX 4, a notable sign of possible immune activation [161], and leaving the consequences of torque-driven LMP in ICD as an open follow-up direction.

Apart from lysosomal accumulation for effective cancer cell death *via* ICD, mitochondria-targeting strategies for cancer cell death and immune activation have also gained immense popularity and have grabbed the attention of researchers on focusing on and developing therapeutic regimes to damage and reduce the membrane potential of mitochondria. In one such study, Jiang et al. [133] came up with a core–shell nanomedicine named MRT involving ZnCoFe2O4@ZnMnFe2O4 grafted with arginyl-glycyl-aspartic acid (RGD) and (3-carboxypropyl) triphenylphosphonium bromide (TPP) to achieve stable dispersion and enable mitochondrial targeting along with high M_s_ aiding in strong AMF-actuated MHT. MRT exhibited negligible cytotoxicity even at high concentrations of 400 μg/mL without AMF, and the same was inverted upon exposure to AMF, with rapid loss in cell viability post-brief MHT of 5 min, with temperatures reaching nearly 50°C. Mitochondrial stress due to MHT elevated the MitoSOX fluorescence signal (90.8%) as affirmed by flow and triggered ICD *via* ER–mitochondria crosstalk involving downregulation of Mfn2 and activation of PERK/eIF2α, resulting in CRT translocation and ATP/HSP70 release. Thus, the rich exposure of DAMPs facilitated the repolarization of macrophages toward M1 (M1: 28.8% M1 and M2: 8.3%).

In a recent but similar study to deteriorate mitochondrial health, Lien et al. [162] formulated a tumor-penetrating solid lipids nanocomplex by loading N-doped carbon dots/mesoporous silica with elesclomol-copper and integrated it with solid lipid/polymer to obtain EsCu@CMS, which exploited pH-responsive charge switching for effective accumulation in lung metastasis site and AMF-enabled cuproptosis *via* delivering elesclomol–Cu payload. The nanosystem colocalized with mitochondria, as affirmed through line scan (confocal microscope), and the mitochondrial membrane potential (ΔΨm) drastically dropped under AMF, resulting in Cyt c-dependent apoptosis. The catechol groups on CMS facilitated antigen capture, thereby enhancing DC activation, resulting in a 150% increase in CD8+ T cells and survival exceeding 60 days in combination with αPD-1, a significant improvement compared with the control group’s 20-day survival.

In a fascinating study leveraging calcium-sensing receptor/caspase-1 signaling, Chen et al. [163] reported MFR-NPs termed MSCH, which, upon exposure to AMF, induced mild hyperthermia and simultaneously released Ca^2+^, thereby polarizing M2 macrophages into an M1 phenotype, resulting in amplified ICD in orthotopic liver cancer models. *In vitro*, flow cytometry analysis to gauge apoptosis in H22 cells revealed a rise in apoptosis accounting for ~45% in the MSCH+AMF-treated group as a consequence of Cyt c-mediated cell death linked to mitochondrial dysfunction. The *in vivo* flow cytometry profiles revealed dramatic polarization into M1 macrophages for MSCH (25.2%) and MSCH+AMF (36.9%), while M2 macrophages decreased drastically to 22.3% and 11.8%, respectively. The major contributors to ICD, such as ATP, HMGB1, and CRT, were elevated, and flow cytometry profiles of tumors extracted from mice indicated effective DC maturation, NK cell activation, and enhanced T-cell infiltration (CD8^+^/CD4^+^) after MSCH+AMF treatment.

While immune system activating magnetic-field responsive nanosystems are majorly scrutinized for their physicochemical properties and ICD-inducing proteins, assessing magnetic properties for optimal delivery of MHT is of utmost significance, for which Wang et al. [164] developed an FVIO nanoring with a size ∼of 57 nm, exhibiting magnetic properties such as Ms = 72 emu/g, SAR = 2285 W/g, and ILP = 10.98 nH·m2/kg. The nanoring exhibited AMF-mediated heating, with a 24°C temperature rise within 10 min, and specifically facilitated CRT translocation from the endoplasmic reticulum. Apart from imparting DAMP signaling, such as CRT, ATP, and HMGB1, MHT facilitated by FVIO was successful in suppressing anti-phagocytic signaling, where *in vivo* SIRPα^+^ within macrophages dropped significantly (15.4% vs 34.7%). More importantly, CD47 expression in nonimmune cells and tumor cells dropped from 51.5% (PBS) to 23.9% (FVIO facilitated MHT) in liver cancer-bearing mice.

Collectively, the aforementioned organelle-targeting MFR-NPs underscore the significance of positioning MFR-NPs into cellular organelles, which not only stresses cancer cells *via* bulk heating but also enhances ICD and reprograms the TME. The next-generation designs of nanomedicines for cancer immunotherapy not only leverage organelle-specific stress but also mediate antigen capture, thereby maximizing immunotherapeutic outcomes. Most importantly, ICD occurs simultaneously with the downregulation of immunosuppressive niches into proactive therapeutic zones to inhibit cancer proliferation. However, the current disadvantage lies in the complexity of modern-day nanoarchitectures, which are of utmost significance for ensuring prolonged stability and safety in biological systems. However, the clinical translation and real-time application of such immunomodulating nanomedicines have limited efficacy, particularly in brain tumors, where barriers such as the BBB, immunosuppression, and a myeloid-enriched niche not only limit nanomedicine accumulation but also impair T-cell function [15,16]. Due to these very barriers, GBM restricts the actions of MFR-NPs, limiting success in therapeutic outcomes, necessitating alternative effective strategies to circumvent the BBB and overcome the hostile neuroimmune microenvironment. In the subsequent section, we therefore elucidated the complications associated specifically with GBM and summarized novel MFR-NP strategies designed to overcome these complications and facilitate effective activation of the innate immune system.

## 7. Glioblastoma microenvironment and magnetic field-actuated nanoarchitectures in its immunotherapy

GBM is the most common form of brain cancer that is not only highly aggressive and malignant but also characterized by infiltrative growth, hypoxic-necrotic core, heterogeneity, and BBB, which make it highly challenging to treat. GBM therapy poses translational hurdles for AMF-responsive nanomedicines, as therapeutic benefit and efficacy must be achieved despite challenges posed by a heterogeneous vasculature, rapid growth, and a hypoxic niche that comprises immunosuppressive myeloid populations that neutralize cytotoxic immunity [165,166]. Moreover, the GBM microenvironment inhibits T-cell function and promotes T-cell exhaustion, limits sustained priming, and thereby hinders the therapeutic efficacy of standard immunotherapeutic strategies, necessitating immunogenic reprogramming [167,168]. The aforementioned GBM-associated complexities dramatically narrow the engineering window for AMF-actuated delivery of therapeutic cargo and activation of MFR-NPs in the tumor, where factors such as magnetic field parameters, eddy current generation, and clinical safety limits (*H*×*f* product also known as Atkinson–Brezovich limit) must be assessed and controlled to balance therapeutic efficacy [169–171]. Accordingly, the following section first discusses GBM-associated physical and immunological constraints for magnetic actuation, then summarizes AMF-responsive nanomedicine design and strategies to circumvent them.

### 7.1. Nanoengineering and biophysical constraints in glioblastoma therapy via magnetic field aided triggering

GBM imposes strict and unique operational challenges on AMF-actuated nanomedicine, as critical factors involving successful delivery, uniform distribution, and intratumoral activation of nanomedicine are constrained by transport physics and clinical safety. Most importantly, differences in BBB integrity and active efflux by GBM cells result in inconsistent intratumoral accumulation of engineered nanoarchitectures, thereby limiting the efficiency of passive accumulation *via* the enhanced permeability and retention (EPR) effect in the central nervous system (CNS) [172–174]. High interstitial fluid pressure, dense and irregular extracellular matrix, impede convection and exacerbate perivascular trapping of nanomedicine, while GBM tumor cells continue to seed and spread malignant cells beyond the reach of radiographic margin, thereby necessitating an effectively designed nanoparticle capable of enhanced penetration and accumulation into the tumor core *via* peritumoral brain [175–177]. When it comes to magnetic field-mediated activation, AMF/high frequency magnetic field (HFMF) variables must comply with patient tolerance and risk of off-target heating and demand coil dimensions that can effectively maximize the delivery of magnetic field at high depth in the subject while simultaneously minimizing the negative effects posed by the generation of eddy-current in the CNS, mandating a strict control over heating and dependable thermometric devices to prevent collateral tissue injury [178].

### 7.2. Landscape of glioblastoma tumor microenvironment

GBM characteristically poses a cold and inflamed microenvironment comprising myeloid cells that predominantly govern the nonmalignant compartment involving both microglia and immunosuppressive macrophages called TAMs, which contribute to a large fraction of TME and adapt frequently to poorly display antigens, regulate TME immune niche to sustain invasion, promote angiogenesis, and resistance to therapy [179]. Adaptive immunotherapy outcomes have shown insignificant therapeutic results due to dysfunction of cytotoxic T-cells, with limited presence of effector T-cells at lesions site and those that enter are usually exhausted due to constant antigen contact and myeloid signal such as IL-10 associated pathways, posing a suppressive effect, thereby hindering the therapeutic efficacy of checkpoint blockade in GBM [168]. This is armored by a cytokine-enriched TME, with immunosuppressive mediators such as IL-10 and TGF-β, and chemokine-mediated expression and signaling that favorably recruit suppressive myeloid cells, thereby inhibiting DC maturation, antigen presentation, and cytotoxic activity of T-cells [180]. Among these, hypoxia and necrosis in GBM reshape the metabolism and stromal signaling to sustain an immunosuppressive environment and restrict T-cell development and function, henceforth running a feed-forward loop to evade immune cells and sustain tumor proliferation and growth [181].

### 7.3. Magnetic field-driven nanomedicines to combat GBM and Potentiate immunotherapy

Magnetic field-triggered nanosystems can be designed and tuned to specifically accumulate in and around GBM for the delivery of therapeutic cargo and disrupt associated immunological barriers by circumventing the BBB, to transduce magnetic-field into localized hyperthermia and eddy current to induce stress in cancer cells, leading to release antigens followed by ICD, and reprogramming myeloid circuits for the effective priming and checkpoint synergy. The sub-section presents unique designs that illustrate the use of MFR-NPs for effective immunotherapy of GBM *via* precise control over MHT, eddy-current, guidance, and immune activation.

In a research to effectively deliver magnetic-field actuated nanosystem with the capability to generate eddy current, Cheng et al. [148] and team designed a nanogold yarn ball with plabociclib-loaded dendrimer camouflaged with RVG for effective and targeted accumulation in brain tumors *via* the spinal cord. The proposed nanosystem RGV@Den[Pb]-GY generated an eddy current upon exposure to AMF (3.2 kW, 1 MHz), which enabled the loosening of tight cancer cell junctions to facilitate deeper payload penetration and enhance T-cell penetration. A release of nearly 17% and 57% of palbociclib-loaded dendrimer upon exposure to AMF for 2 min and 10 min, respectively. The RGV@Den-GY nanomedicine was highly biocompatible toward ALTS1C1 cells at a concentration of 20 µg/mL, with the cell viability accounting for >90%. On the contrary, the same nanomedicine for the same concentration posed a significant cytotoxic effect upon exposure to AMF. The *in vivo* assays revealed a survival of more than 50% mice over a period of 60 days for orthotopic GBM-bearing mice treated with RGV@Den[Pb]-GY+AMF, which can be attributed to a robust immune response, as affirmed by staining of brain sections for CD4 and CD8 markers.

While MHT mediated cancer has been the major focus for cancer therapy, in an simple yet interesting study, Cui et al. [182] reported a magnetic silica–PLGA nanoparticles loaded with DOX and paclitaxel (PTX), which were further modified with transferrin for targeted delivery of the nanosystem to glioma, along with an external magnet (neodymium-iron-boron, 0.1T) for retention of nanomedicine. Confocal imaging confirmed significant uptake and retention of nanoparticles for the magnetic-field-treated group compared with the control, and the results were consistent under in-vivo conditions, where bioluminescence imaging confirmed significant retention of nanoparticles along with acceptable body weight and histological examination results, thereby positioning magnetic field-aided retention and targeting as a delivery amplifier rather than MHT and ICD. Wu et al. [183] built a dual redox-responsive PCL-SeSe-PEG nanocarrier for the efficient delivery of fingolimod into GBM, antagonizing S1P1 to disrupt endothelial barrier and tight-junction proteins, thereby allowing paracellular entry of high-SAR Zn/Co-doped iron-oxide nanoclusters. The design delivered VER-155008, a HSP70 inhibitor that sensitizes tumors to MHT, and was tested in GL261 and U87 models, which indirectly supports immunotherapy by localized heating stress.

In an attempt to induce ICD, Liu et al. [184] fabricated an ultrasmall magnetic field-responsive nanosystem comprising Mo_0.2_ Fe_2.98_ O_4_ that was further coated with CeO_x_ followed by modification with folic acid for active targeting. The proposed nanosystem also employed static magnetic guidance to enhance BBB permeability. Upon irradiation of AMF, the nanosystem named MFCF exhibited MHT, ROS generation, and depleted GSH in combination with hypoxia relief to activate ferroptosis. This immunotherapeutic regimen facilitated the repolarization of M2 macrophages to the M1 phenotype under controlled, confined magnetic field-mediated MHT.

While inorganic magnetic nanosystems have been modified with ligands and cancer receptor-binding molecules, Ceccarelli et al. [185] reported a simple yet effective lipid-based nanovector that was doped with IONPs to specifically engage with microglia and its biology simultaneously with AMF-mediated thermal transduction to elevate the intracellular Ca^2+^ levels that result in reprogramming of macrophages to M1-like. This repolarization of macrophages was further corroborated by the upregulation of CD40/CD86, along with the release of IL-6, IL-8, and TNF-α. Moreover, assays conducted using conditioned media from groups treated with LMNVs+AMF impaired GBM cell-proliferation and exhibited hallmarks such as HMGB1 and CRT translocation that are specific to ICD.

**Figure 8.**
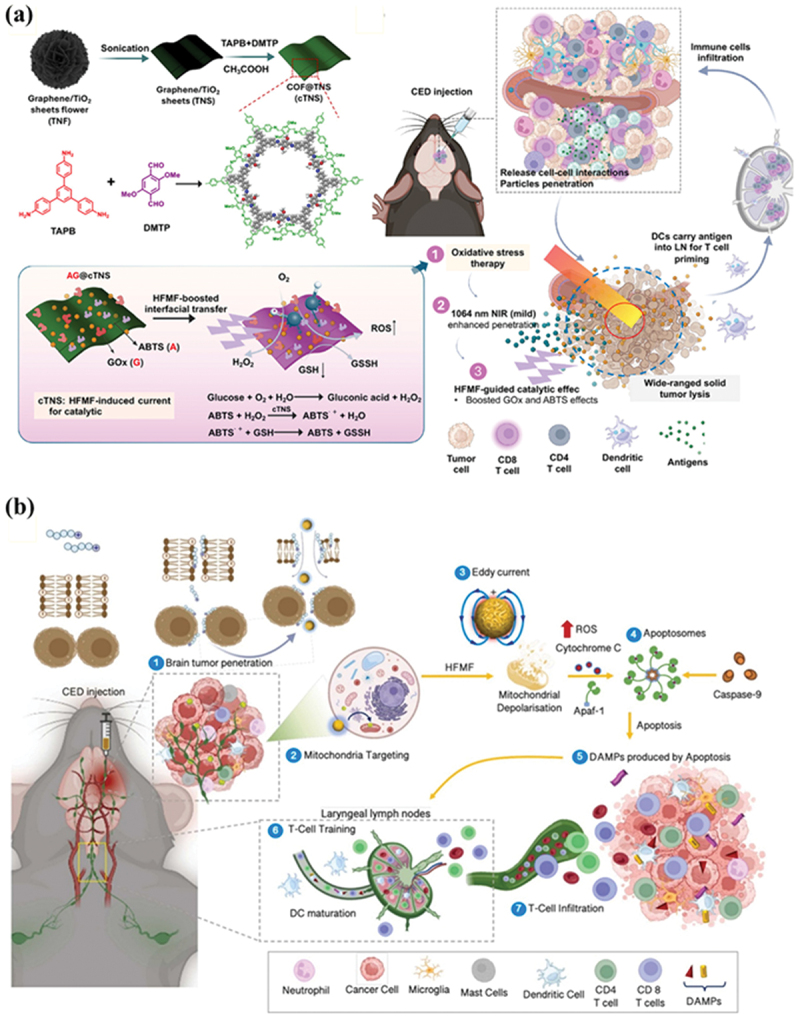
(a) Scheme represents the fabrication of AG@cTNS and its application in combination therapy involving NIR-II mediated ROS production and HFMF induced amplified catalytic activity resulting in effective DC priming and infiltration of immune cells. Reproduced with permission from [186]. Copyright 2025, John Wiley & Sons. (b) Illustration depicting the wireless charging of mitochondria-targeting WINA, resulting in the generation of an eddy current, which, in consequence, results in the reduction of membrane potential and triggers apoptosis, leading to DAMPs-mediated DC maturation and T-cell infiltration. Reproduced with permission from [20]. Copyright 2025, Elsevier Ltd.

In a fascinating study involving the combination of PTT and MHT, Huynh et al. [186] and colleagues fabricated a cascade-responsive “catalytic nanosponge” from oxygen-deficient graphene/TiO_2_ nanosheets and further surface modified with covalent organic framework (COF) for the effective co-loading of GOx and ABTS for GSH exhaustion and generation of ROS (^1^O_2_ and •OH radicals). The nanomedicine AG@cTNS facilitated GBM immunotherapy by capturing intratumoral antigens and remotely activating immune cells (Figure 8(a)). The GOx loaded in AG@cTNS actively participated in augmenting H_2_O_2_ levels from intracellular glucose upon exposure to NIR-II (1064-nm) and HFMF, followed by accelerated production of singlet oxygen (^1^O_2_) and hydroxyl (•OH) radical *via* NIR photon-induced electron hole separation and magnetic field-actuated catalysis. The ABTS is oxidized to its cationic form, ABTS•+, which drives GSH depletion, exacerbates oxidative stress, and facilitates ICD. The proposed nanomedicine was evaluated for anti-cancer effects in ALTS1C1, U87MG, and GL261 cells, where AG@cTNS, in combination with HFMF-treated groups, augmented GBM cell killing *via* ROS and disrupted 3D spheroids. In glioma-bearing mice, the nanomedicine was delivered through convection-enhanced delivery (CED) to overcome the hindrances posed by the BBB. The immunotherapeutic outcome assessed through flow cytometry of the lymph node affirmed a significant increase in mature DCs (CD80/CD86), along with boosted CD4/CD8 cells. Moreover, the survival results revealed 100% survival for GBM-bearing mice treated with AG@cTNS+NIR-II/HFMF+anti-PD-1.

While efforts combining PTT and MHT for GBM immunotherapy have shown promising outcomes, the simple delivery of checkpoint inhibitors, antibodies, and small-molecule agonists has also attracted considerable attention and yielded significant reprogramming of the GBM immune TME. In one such effort, Zhang et al. [187] presented a study involving lipid nanoparticles loaded with anti-CD47/PD-L1 that simultaneously block both innate CD47 and effector PD-L1, delivering the STING agonist diABZI to reprogram the myeloid circuit. Although the proposed system is not magnetic-field-actuated, the study is significant for rewiring the GBM immunological TME to regulate T-cell infiltration in synergy with radiotherapy. The immunotherapeutic outcomes revealed cytotoxic T-cell infiltration, with a sixfold increase in CD8+ T-cell infiltration in GBM, thereby indicating that innate circuit connectivity can be a dominant factor in GBM.

In a similar approach, circulating around the lipid nanoparticles, Pucci et al. [188] developed a design in which lipid nanovectors were functionalized with angiopep-2 and encapsulated SPIONs and Nutlin-3a to facilitate BBB transcytosis and GBM MHT. The nanosystem not only exhibited conventional MHT driven by AMF but was also linked to LMP to initiate caspase-dependent apoptosis. A BBB/GBM microfluidic co-culture device was employed to scrutinize GBM targeting, and the same exhibited exceptional results with significant targeting capability. Although the study primarily focused on *in vitro* assays, immunofluorescence revealed HSP release, which is linked to cancer cell stress-mediated ICD *via* priming. In a fairly recent study, Rivera et al. [189] reported a design in which iron oxide nanoparticles were intracranially delivered to trigger MHT upon exposure to AMF, along with the chemodrug temozolomide and radiation. The tumor models treated with MHT+CRT showed a significant reduction in the tumor burden and improved the overall survival. Moreover, elevated levels of DNA damage response and HSP were observed alongside microglial activation and increased CD4^+^ population. The results of this combination therapy remain valid despite stress-amplified antigen release and immune activation.

In addition to MHT, the generation of eddy currents has played a crucial role in inhibiting GBM progression and activating the innate immune system. In one such effort to effectively trigger immune activation, Moorthy et al. [20] developed a wirelessly chargeable nanocomplex, termed WINA, using gold nanoparticles that were loaded with and conjugated to ONC201 and polymerized GSH, comprising triphenylphosphine, respectively (Figure 8(b)). The proposed nanosystem, WINA, was delivered intracranially *via* CED, and its positive surface charge facilitated mitochondrial targeting and reduced ΔΨm, resulting in apoptosis. The immunotherapeutic results, as affirmed through the extraction of lymph nodes, revealed a significant elevation in the T-cells, where it increased to nearly 40.9% in ONC201@WINA+HFMF treated groups, which was further backed by a 5.5% maturation in DCs along with antigen capture.

With substantial improvements in MFR-NP design to circumvent the BBB and immunological barriers, magnetic field-actuated nanomedicines have the potential to accumulate in GBM and elicit an immune response to support effective GBM therapy. The primary use case of MFR-NPs in the treatment of GBM is their capability to wirelessly generate hyperthermia and eddy current upon exposure to a magnetic field. Secondly, AMF-responsive nanosystems enable deep-tissue actuation across the BBB, which not only facilitates imaging and surveillance of the GBM but also aids in reprogramming the neuro-immunological niche. However, the future and its success still depend solely on the critical aspect of the Atkinson–Brezovich safety limit in human-scale applications. Most importantly, substantial clinical trials are needed to further evaluate their therapeutic efficacy, systemic toxicity, optimal MHT dose, clinical safety limits, and active targeting capabilities, to reduce the risk of side effects from dissociated metallic ions and off-target delivery of inorganic nanomaterials, along with strong results confirming their preferential accumulation in GBM.

## 8. Translational bottlenecks and roadmap for application of AMF-Responsive nanomedicines in cancer immunotherapy

While AMF-responsive nanomedicines have shown promising outcomes in mouse models, their upscaling and clinical translation are limited by the Atkinson–Brezovich safety limit. More importantly, several assumptions in this field require rigorous scrutiny before a preliminary clinical trial. The most critical factor is magnetic field variables (H×f) to prevent heating and damage to healthy tissues [169–171]. Circumventing this hurdle requires advanced human-sized coils that are specifically designed to project a magnetic field into deep-seated tumors with minimal collateral surface exposure and deterioration [4,22]. The reliance on passive accumulation needs to be reduced, and innovative design strategies that enable targeted accumulation and image-guided navigation of AMF-responsive nanosystems should be adopted to enable real-time, quantitative 3D mapping of nanoparticles, ensuring precise positioning prior to AMF irradiation to reduce collateral damage of healthy tissue and improve biocompatibility [190]. Apart from the aforementioned tactics, meticulous engineering and scrutiny are still needed. Accomplishing synergistic control over bulk heat, electron transfer, and inducing an immune response still remains a complex challenge. Also, exposure to high thermal doses increases the risk of thermotolerance. However, the emergence of magnetocatalytic and magnetoelectric nanosystems has offered heat-free techniques to trigger ICD via ROS-mediated stress and to disrupt electron transport and induce DNA damage in cancer cells, even in the presence of barriers like the BBB [186]. Moreover, standardizing laboratory protocols for large-scale production, along with rigorous quality control and safety verification, is of utmost significance to prevent systemic toxicities arising from degraded inorganic products of nanomaterials. Thus, developing an integrated real-time feedback loop with next-generation smart nanoarchitectures is the demand for the future of the field to transition from conceptual animal experiments to leap forward with more reliable and durable therapeutic nanomedicines to improve survival outcomes in patients suffering from cancer.

### 8.1. Metabolic fate, long-term stability, and biocompatibility

The clinical use of MFR-NPs for cancer therapy hinges solely on their long-term biocompatibility and systemic clearance. While SPIONs are tested and proven to be highly biocompatible and biodegradable as they undergo lysosomal degradation in the hepatic system and spleen, the recent evolution of MFR-NPs into hybrid nanoantennae introduces a new challenge associated with their stability and deterioration [7]. In spite of significant advancements being made in biofunctionalization of MFR-NPs, achieving effective intra-tumoral accumulation, uniform distribution, and uniform heating in heterogeneous tumors like GBM in the presence of the BBB remains a challenge. Advanced nanosystems, such as MOFs, can degrade in serum as phosphate and albumin compete for metal ions chelated by organic linkers of MOFs, thereby leading to premature cargo release and structural disintegration [191]. Also, the doping of magnetic elements such as ‘Co’ and ‘Ni’ into MFR-NPs is limited by their high genotoxicity and the increased risk of systemic toxicity from unintended leaching. For nanosystems and carriers that are non-biodegradable, such as ‘Au’ or carbon shells, clinical safety needs strenuous testing over size-dependent renal clearance (typically <6–8 nm) to prevent nanoparticles accumulation in the immune cells (monocytes and macrophages) responsible for engulfing and clearing the foreign particles and inhibiting inflammatory response [192]. Thus, to address these metabolic complications, green synthesis of nanomedicines and coating them with biocompatible, anti-fouling polymers (e.g., PEG) is one of the most proven tactics for ensuring the effectiveness and clinical safety of MFR-NPs [51]. While inorganic MFR-NPs have shown promising biocompatibility, their long-term fate, slow degradation, and potential toxicities within the human body need to be rigorously scrutinized prior to commercialization.

## 9. Conclusion

The scenario of cancer therapy is undergoing a drastic shift involving a transition from conventional bulk MHT to cancer systemic interventions involving spaciotemporally controlled techniques. MFR-NPs have evolved as a promising candidate that offers an advantage over conventional therapeutic strategies involving the transduction of magnetic field into thermal energy alongside controlled drug release, catalytic reactions, and triggering the innate immune system. As discussed in this review, the underlying physics governing MFR-NPs, ranging from Néel and Brownian relaxation to hysteresis loss, enables meticulous engineering for effective cancer therapy. Looking further, the advancement of MFR-NP-based immunotherapy nanomedicine lies in the engineering and further development of smart and hybrid nanoarchitectures. These smart nanosystems must not only be capable of circumventing the BBB in the case of GBM but also specifically target and interact with immune cells for effective activation of the immune system and trigger ICD. The synergistic outcomes of MHT-aided ICD and checkpoint blockade induced by AMF-actuated MFR-NPs must be optimized to achieve a robust antitumour immune response and memory. The future research must focus on bridging the gap between physics and immunooncology. The development of next-generation magnetic field-responsive smart nanoarchitectures is of the utmost importance to counter cancer and associated barriers for a sustained immune response with durable therapeutic and survival outcomes in cancer patients.

## Article highlights

- Magnetic-field-responsive nanoparticles (MFR-NPs) have advanced from simple hyperthermia agents to multifunctional platforms that combine catalytic therapy with electrically driven effects under alternating magnetic fields (AMFs).
- Precise control over nanoparticle composition, size, and morphology significantly enhances specific absorption rate (SAR), magnetothermal efficiency, and magneto-catalytic activity.
- AMF exposure enables localized eddy currents and voltage gradients in conductive nanostructures, facilitating Joule heating and remote electrochemical stimulation.
- MFR-NPs allow spatiotemporal drug release, deeper tumor penetration, and electrically regulated intracellular redox processes, improving therapeutic precision.
- Magnetically triggered catalytic systems promote cuproptosis and immunogenic cell death, enhancing anti-tumor immunity and showing strong promise in treating glioblastoma and metastatic cancers.
